# CHCHD2 mitigates age-associated proteostatic collapse by maintaining homeostasis of the mitochondria-lysosome axis

**DOI:** 10.1007/s00401-026-03093-4

**Published:** 2026-10-13

**Authors:** Teresa R. Kee, Brianna M. Masters, Khoi D. Tran, Hanna Jeon, Ma Long, Grace Xu, Sophia A. Khan, Edwin Vázquez-Rosa, Preethy S. Sridharan, Yeojung Koh, Hisashi Fujioka, Dale Chaput, Xinming Wang, Xingyu Zhao, Andrew A. Pieper, David E. Kang, Jung-A A. Woo

**Affiliations:** 1 Department of Pathology, School of Medicine, Case Western Reserve University, Cleveland, OH USA; 2 Department of Psychiatry, School of Medicine, Case Western Reserve University, Cleveland, OH USA; 3 Department of Neuroscience, School of Medicine, Case Western Reserve University, Cleveland, OH USA; 4 Institute for Transformative Molecular Medicine, School of Medicine, Case Western Reserve University, Cleveland, OH USA; 5 Electron Microscopy Core Facility, School of Medicine, Case Western Reserve University, Cleveland, OH USA; 6 Department of Molecular of Medicine, USF Health College of Medicine, Tampa, FL USA; 7 Department of Cell Biology, Microbiology, and Molecular Biology, University of South Florida, Tampa, FL USA; 8 Brain Health Medicines Center, Harrington Discovery Institute, University Hospitals Cleveland Medical Center, Cleveland, OH USA; 9 Geriatric Psychiatry, Geriatric Research Education and Clinical Center, Louis Stokes VA Medical Center, Cleveland, OH USA; 10 Louis Stokes VA Medical Center, Cleveland, OH USA

**Keywords:** CHCHD2, Lewy body disorders, Mitochondria-lysosome axis, Aging

## Abstract

Mutations in coiled-coil-helix-coiled-coil-helix domain containing 2 (CHCHD2), including the Parkinson’s disease (PD)-linked CHCHD2^T61I^ mutation, are associated with Lewy body disorders (LBDs) by unknown mechanisms. Here, we demonstrate that CHCHD2^T61I^ exacerbates age-related brain pathologies, including axonal degeneration and cognitive deficits, by disrupting the mitochondria-lysosome axis. Specifically, CHCHD2^T61I^ binds and reduces cathepsin B (CTSB) and cathepsin L (CTSL) levels, inducing lysosomal membrane permeability and impairing α-synuclein fibril clearance. We also demonstrate that human CHCHD2 declines in the brain with age, while expression of wild-type CHCHD2 safeguards against age-related neuroinflammation, dopaminergic neuron loss, insoluble protein aggregation, and structural deterioration of mitochondria and lysosomes. These findings, supported by observed reductions in CTSB and CHCHD2 in human LBD brains, establish CHCHD2 as a key regulator of neuronal resilience to aging and proteostatic collapse via the mitochondria-lysosome axis. Preventing age-associated CHCHD2 decline offers a therapeutic strategy to preserve brain health throughout aging.

**Supplementary Information:**

The online version contains supplementary material available at https://doi.org/10.1007/s00401-026-03093-4.

## Introduction

Age-related accumulation of α-synuclein (αSyn) in Lewy bodies (LBs) is a central pathological hallmark of Lewy body disorders (LBDs), including Parkinson’s disease (PD) and dementia with Lewy bodies [34]. Mitochondrial and lysosomal dysfunction are key drivers of LBD pathogenesis, intersecting with both αSyn pathology and normal brain aging [6, 29, 39, 52, 54, 59]. Pathological αSyn binds to mitochondrial translocase of outer mitochondrial membrane 20 (TOM20), impairing mitochondrial protein import [17] and complex I activity [40], features prominently observed in LBD patient brains [12, 24, 57]. Age-related declines in lysosomal-mediated mitophagy [5, 58, 59], exemplified by mutations in *PINK1* and *PARKIN* [50, 60], and variants in lysosomal genes (*GBA*, *ATP13A2*, *TMEM175*, *CTSB,* and others) linked to PD risk [63] further exacerbate disease mechanisms. Pathological αSyn also permeabilizes lysosomal membranes [21, 31, 77], disrupting lysosomal exocytosis and promoting lysosomal rupture that further propagate misfolded αSyn aggregation [21, 31, 77]. Despite these insights, the mechanistic interplay between mitochondrial-lysosomal axis dysfunction and LBD progression remains poorly defined.

The mitochondrial intermembrane space (IMS) protein coiled-coil-helix-coiled-coil-helix domain containing 2 (CHCHD2, abbreviated as D2) regulates electron transport chain function, cristae integrity, and apoptosis [32, 42, 51, 55]. PD-linked D2^T61I^ and D2^R145Q^ proteins co-segregate with autosomal dominant PD [23, 67, 78], while over 20 additional D2 mutations are associated with LBDs [32]. Patients with D2^T61I^ exhibit substantia nigra neurodegeneration, L-DOPA-responsive motor deficits, and pathologies extending beyond LBDs, including neurofibrillary tangles and amyloid-β plaques, implicating D2^T61I^ in multifaceted neurodegenerative disease [27].

We previously established neuron-specific transgenic mice expressing human D2^T61I^, which recapitulate PD features such as synaptic loss, neuroinflammation, mitochondrial aggregation, dopaminergic neurodegeneration, and αSyn accumulation [33]. These findings were validated in independent D2^T61I^ models [19, 38, 71]. Here, we introduce a CHCHD2^WT^ (D2^WT^) mouse model that demonstrates neuroprotection against age-related neuroinflammation, dopaminergic neuronal loss, proteinopathy, and mitochondrial-lysosomal axis deterioration. Through new proteomic and interactome approaches, coupled with mechanistic studies and analysis of postmortem human tissue, we reveal that D2^T61I^ and age-dependent D2^WT^ reduction disrupt the mitochondria-lysosome axis by depleting lysosomal proteases, driving LBD-like protein aggregation, neurodegeneration, and cognitive impairment. Our findings position D2 as a pivotal regulator of neuronal resilience, with therapeutic implications for protecting the brain from age-related deterioration.

## Materials and methods

### Mouse models

All animal experiments comply with current United States law and were approved by the Institutional Animal Care and Use Committee (IACUC) at Case Western Reserve University (CWRU). IACUC and Institutional Biosafety Committees at CWRU have also approved that all the methods used in this study were performed in accordance with the relevant guidelines and regulations. Wild type (WT) C57BL/6 J and hemizygous transgenic mice expressing CHCHD2^T61I^ or CHCHD2^WT^ were bred in the C57BL/6 J background. Both transgenic strains have been deposited at The Jackson Laboratory (strain numbers: 039978 and 039979). Mice were kept at standard vivarium conditions with food and water supplied ad libitum with a 12-h light/dark cycle. Transgenic mice were generated as previously described [33, 43]. To generate transgenic mice expressing human CHCHD2^T61I^ or CHCHD2^WT^, a transgenic expression construct containing the mouse *Prp* promoter (MoPrP.Xho) was obtained from Dr. David Borchelt (University of Florida, FL, USA). This construct has previously been used to create other mouse models of neurodegeneration, such as the α-synuclein^A53T^ [36], PS19 tau^P301S^ [79], and APP/PS1 [28] transgenic models. Flag-CHCHD2^T61I^ or Flag-CHCHD2^WT^ was subcloned into the XhoI site of the MoPrP.Xho vector and sequenced for validity. A validated transgenic plasmid was sent for embryo injection to the University of Utah Gene Targeting Core (Salt Lake City, Utah, USA). The transgenic construct was microinjected into at least 200 fertilized C57BL/6 J embryos and implanted into pseudo-pregnant mice. Genomic DNA isolated from tail snips from over 100 offspring was used for genotyping by PCR with the following primer sequences for human CHCHD2: forward primer, 5’-CAG CAG CCT TGC CTC TAT GA-3’ and reverse primer, 5’-GTA CAA GAA AGT TGG GTA GGC CAA T-3’. Positive founders were bred with C57BL/6 J mice for a minimum of 3 generations prior to characterization. To quantify expression of the CHCHD2^WT^ transcript compared to endogenous expression of CHCHD2, total RNA isolated from C57BL/6 J, CHCHD2^T61I^, and CHCHD2^WT^ mice were subjected to qRT-PCR using primers common to both mouse and human CHCHD2: forward primer, 5’-ATG CCG CGT GGA AGC CGA AG-3’ and reverse primer, 5’-CCT GAG GCT CCT GGT AAG TG-3’.

### Mouse behavior testing

#### Novel object recognition

**Fig. 1:**
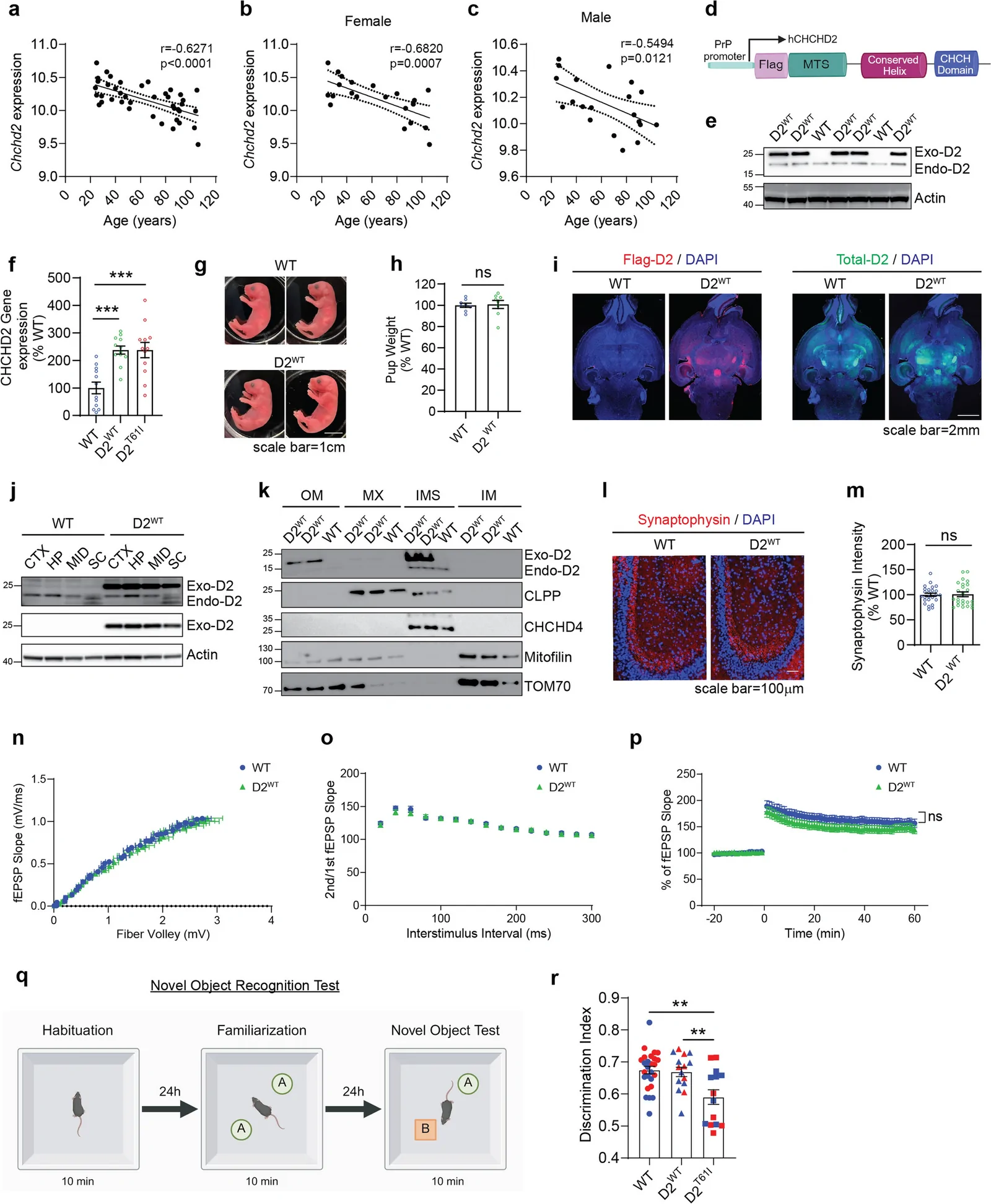
Age-related decline in *Chchd2* expression in human brain and normal phenotypes in CHCHD2^WT^ mice. **a** Correlation of human frontal cortex *Chchd2* gene expression (log_2_ transformed) and biological age in both sexes (Pearson’s correlation, r = − 0.6271, p < 0.0001, n = 41), **b** in females (Pearson’s correlation, r = − 0.6820, p = 0.0007, n = 21), and **c** in males (Pearson’s correlation, r = − 0.5494, p = 0.0121, n = 20). **d** Schematic representation of the human Flag-CHCHD2^WT^ transgene driven by the *mPrp* promoter. Created with BioRender.com. **e** Representative immunoblots for CHCHD2 and actin from whole brain extracts of CHCHD2^WT^ P0 pups and WT littermates. **f** RT-qPCR analysis of CHCHD2 gene expression in the cortex of 12-month-old WT, CHCHD2^WT^ and CHCHD2^T61I^ mice (one-way ANOVA; post hoc Tukey, ***P < 0.001, *n* = 4 mice/genotype with 3 replicates). **g** Representative images of WT and CHCHD2^WT^ P0 pups. **h** Quantitative analysis of whole-body weights from P0 pups (*t* test, *ns* not significant, n = 8 mice/genotype). **i** Representative images of horizonal whole brain slices stained for M2/Flag (red) and CHCHD2 (green) to visualize exogenous and total CHCHD2, respectively, in 12-month-old mice. **j** Representative immunoblots for CHCHD2, M2/Flag, and actin from RIPA-soluble lysates from cortex (*CTX*), hippocampus (*HP*), midbrain (*MID*), and spinal cord (*SC*) from 18-month-old CHCHD2^WT^ and WT littermates. **k** Mitochondrial sub-fractionation representative immunoblots for CHCHD2, matrix (*MX*) marker CLPP, intermembrane space (*IMS*) marker CHCHD4, inner membrane (*IM*) marker mitofilin and outer membrane (*OM*) marker TOM70 from whole brains of 3- to 4-month-old CHCHD2^WT^ and WT mice. **l** Representative images of hippocampus tissue from 12-month-old CHCHD2^WT^ and WT littermates immunostained for synaptophysin (red) and DAPI (blue). **m** Quantitative analysis of synaptophysin intensity (*t* test, *ns* not significant, n = 4–6 images/mouse from 4 mice/genotype). Electrophysiological recordings, including **n** input–output, **o** paired-pulse facilitation, and **p** long-term potentiation from the hippocampus of 10-month-old CHCHD2^WT^ and WT littermates (two-way ANOVA; post hoc Dunnett’s, *ns* not significant, n = 25 WT slices and 32 CHCHD2^WT^ slices from 4 mice/genotype). **q** Schematic representation of the experimental flow for novel object recognition (*NOR*) testing. Created with BioRender.com. **r** Quantitative analysis of NOR testing performed on 18-month-old WT, CHCHD2^WT^, and CHCHD2^T61I^ mice (one-way ANOVA; post hoc Tukey, **P < 0.005, n = 26 WT, 15 CHCHD2^WT^, and 14 CHCHD2^T61I^). Males are blue, and females are red

A schematic representation of this test is in Fig. 1q. Prior to testing on each day, mice were habituated to the testing room for 30 min. On day 1, mice were placed in the center of an empty test arena and allowed to habituate to the environment for 10 min. On day 2, mice were placed in the center of the arena with two identical objects at opposite corners and allowed to explore and familiarize themselves with the objects for 10 min. On day 3, one object was replaced with a novel object. Mice were placed in the center of the arena and allowed to explore the objects for 10 min. The day 3 test session was recorded with an overhead camera. After testing, the recording was used to measure the time spent exploring each object. Mice were considered exploring if they were sniffing or touching an object. Time spent on top of an object was not counted as exploring. The discrimination index was calculated as the time spent exploring the novel object divided by total time spent exploring both objects.

#### Open field testing

Prior to the beginning of the test, mice were habituated to the testing room for 30 min. Mice were placed into the center of the test arena and recorded for 10 min using an overhead camera. Activity was quantified using AnyMaze software (Stoelting, USA).

### DNA constructs and stable cell generation

#### Cloning strategies

HA-CHCHD2^WT^ and HA-CHCHD2^T61I^ were cloned into the pcDNA3.1 backbone with KpnI and XhoI. LAMP1-mGFP (#34831) was obtained from AddGene. We received the pcDNA5-pDEST- FLAG-MTS(AIFM1)-BirA construct from Dr. Hana Antonicka’s lab, which was described previously [3]. The CHCHD2 coding sequence was amplified from the HA constructs and cloned into pcDNA5-pDEST-FLAG-MTS(AIFM1)-BirA with AscI and XhoI (forward primer 5’ TTTTT GGC GCG CCA ATG CCG CGT GGA AGC CGA AG 3’ reverse primer 5’ TTTTT CTC GAG TTA GGC CAA TCC GTT TGC AAG T 3’). All constructs were verified by sequencing prior to use.

#### Lentivirus production

The pLenti-III-HA construct was obtained from ABM (LV022). MTS-BirA (empty vector), MTS-BirA-CHCHD2^WT^, and MTS-BirA-CHCHD2^T61I^ plasmids were cut with HindIII, the 5’ overhang was blunted with Hot Start (New England Biolabs), and linearized plasmids cut with XhoI. Then they were inserted into the pLenti-III-HA construct with EcoRV and XhoI. The constructs were verified by sequencing prior to packaging. Lentivirus was packaged as previously described [74]. Briefly, MTS-BirA-CHCHD2^WT^ and MTS-BirA-CHCHD2^T61I^ lentivirus vectors were co-transfected with pVSVG and Pax2 using FuGENE HD in HEK293 cells overnight. The medium was removed and replaced with serum-free medium the next day. After 72 h of incubation, the medium was collected and centrifuged at slow speed to remove cell debris and filtered through a syringe filter (0.2–0.45 μm).

#### Stable cell generation

HT22 mouse hippocampal cells were transduced with MTS-BirA, MTS-BirA-CHCHD2^WT^, or MTS-BirA-CHCHD2^T61I^ lentivirus with 10 μg/mL polybrene (Santa Cruz Biotechnology, sc-134220) for 48 h. Stable cells were selected with 10 μg/mL of puromycin (ThermoFisher, A1113803) until the negative control plate of non-transduced HT22 cells fully died. Stable cells were maintained in 5 μg/mL of puromycin.

### Recombinant protein production

#### Cloning

CHCHD2^WT^ and CHCHD2^T61I^ were subcloned into the pET22B( +) vector with HindIII and XhoI. Our vectors p3Xflag-CHCHD2^WT^ and p3Xflag-CHCHD2^T61I^ were used as templates. Constructs were transformed into Rosetta *E. coli* to express the recombinant proteins. (Primers F: 5’-TTTTTAAGCTTATGCCGC GTGGAAGC-3’ R: 5’-TTTTTCTCGAGGGCCAATCCGTTTGC-3’).

#### Recombinant protein expression and purification

Rosetta cells transformed with pET22B-CHCHD2^WT^ and pET22B-CHCHD2^T61I^ were cultured at 37 °C, and 500 μM IPTG was added to induce the recombinant protein expression at 25 °C overnight. 1 L of cultured *E. coli* was collected, centrifuged at 3500 r.p.m. (Eppendorf 5424R) for 15 min, the supernatant removed, the cells lysed with lysis buffer (20 mM Tris pH 8.0, 150 mM NaCl, 20 mM imidazole, 0.5% triton) with protease inhibitors, sonicated for 5 min, at 60% amplitude, incubated on ice for 15 min, then centrifuged at 15,000xg for 15 min. 300 μL of Ni sepharose beads (Cytiva) were added to the supernatant, rotated at 4 °C for 1 h, then centrifuged at 700xg for 1 min. Beads were washed with washing buffer (20 mM Tris pH 8.0, 500 mM NaCl, 20 mM imidazole) at 4 °C for 15 min 3 times. Beads were eluted with 300 μL of elution buffer (20 mM Tris pH 8.0, 150 mM NaCl, 200 mM imidazole, 1 mM DTT), rotated for 30 min at 4 °C, then centrifuged at 700xg for 1 min. Proteins were desalted with 10 K Amicon Ultracentrifugal filters (Millipore) and dilution buffer (20 mM Tris pH 8.0, 150 mM NaCl, 1 mM DTT).

#### Preparation of CHCHD2 aggregates

20 μL of recombinant CHCHD2^WT^ or CHCHD2^T61I^ protein (100 μM) was vibrated at 1500 r.p.m. for 16 h at 37 °C to form aggregates. The same amount of protein was kept at 4 °C as negative controls.

### Cell lines, immunocytochemistry, co-immunoprecipitation, and harvesting

#### Cell culture

HT22 mouse hippocampal cells (David Schubert, Salk Institute, La Jolla, CA) and HEK293T human embryonic kidney cells (ATCC, CRL-3216) were cultured in Dulbecco’s modified Eagle’s medium (Gibco, 11-965-092) supplemented with 10% fetal bovine serum (FBS) (Sigma-Aldrich, 12306C), 1% penicillin/streptomycin (P/S) (Gibco, 15140–122), and BM-cyclin (Roche, 10799050001). Cells were kept at 37 °C and 5% CO_2_. MTS-BirA HT22 stable cells were maintained in 5 μg/mL puromycin (ThermoFisher, A1113803) for cell selection.

#### Transfection

DNA plasmids were transiently transfected in HT22 or HEK293T cells using lipofectamine 2000 (Invitrogen, 116688500) or FuGENE® HD (Promega, E2311) and Opti-MEM (Gibco, 31985–070). When using lipofectamine 2000, 4–6 h post-transfection the medium was replaced with a new complete medium. Cells were grown for 48 h after transfection before experimental assays.

#### Immunocytochemistry and proximity ligation assays (PLA)

Immunocytochemistry and PLA were performed as previously described [11, 20]. Cells were washed twice with 1× PBS and fixed with 4% paraformaldehyde (Acros Organics, 41678–5000) for 20 min at room temperature. Fixed cells were blocked with 3% normal goat serum (Vector Laboratories, S-1000) and permeabilized with 0.2% triton (Bio-Rad, 1610407) in PBS at RT for 1 h. Indicated primary antibodies were applied overnight at 4 °C, followed by Alexa fluor secondary antibodies and DAPI (Thermo Scientific, 62248) for 45 min at RT, and mounted with Fluromount-G (Thermo Fisher Scientific, 00-4958-02). Vacuoles were quantified by counting the number of LAMP1-positive vacuoles per field (defined as large vacuolar structures surrounded by LAMP1-GFP without DAPI or CHCHD2), then normalizing to the percent per field in the vector control transfected condition. For lysosomal staining, cells were incubated with LysoTracker Red (Thermo Fisher Scientific, L7528) at a final concentration of 1 μM for 30 min before fixation and PLA. For PLA, overnight incubation with primary antibodies at 4 °C was followed by incubation of the Duolink^®^ In Situ PLA^®^ secondary antibody probes (Sigma-Aldrich, DUO92004 and DUO92002) at 37 °C for 1 h. Duolink^®^ In Situ PLA^®^ ligation and amplification reagents (Sigma-Aldrich, DUO92008, DUO92014, or DUO92013) were incubated at 37 °C for 30 min and 100 min, respectively, before being mounted with the Duolink^®^ In Situ PLA^®^ Mounting Medium with DAPI (Sigma-Aldrich, DUO82040). Each experimental replicate included samples with only one primary or only one probe as negative controls. Slides were stored at −20 °C. In PLA experiments where no fluorescent marker was present, mApple was transfected at a 1:5 ratio as a marker of transfected cells, and only mApple-positive cells were included in the quantitative analysis. A list of all antibodies used can be found in Table 1.

#### Cell harvesting

RIPA-soluble extraction from cultured cells was performed as previously described [11]. Briefly, cells were washed with 1× PBS 3 times on ice, followed by the addition of RIPA lysis buffer (50 mM Tris–HCl pH 7.4, 150 mM NaCl, 2 mM ethlenediaminetetraacetic acid (EDTA), 1% NP-40, 0.1% sodium dodecyl sulfate) with protease and phosphatase inhibitors (GeneDEPOT, P3200-005 and P3100-010). Cells in RIPA were scraped from plates and spun down at 4 °C and 15,000 r.p.m. (Eppendorf 5424R) for 15 min to separate the RIPA insoluble pellet. The supernatant was transferred to a new tube, the pellet washed with 100 μL of 1× RIPA with inhibitors, centrifuged at 4 °C and 15,000 r.p.m. (Eppendorf 5424R), and the wash removed. The pellet was then sonicated in sample buffer part 1 (10% sodium dodecyl sulfate, 250 mM Tris–HCl pH 6.8) in a room-temperature water bath sonicator (QSonica, Q800R3) in two-minute intervals at 50% amplitude with a pulse lasting two seconds then resting for one second before pulsing again. Sonication was repeated until the sample was fully homogenized; this is the RIPA-insoluble sample. A BCA kit (BCA protein assay, Pierce, 23225) and a Biotek microplate reader were used to determine protein concentration. Soluble and insoluble samples were equalized to the same concentration. 4× sample buffer (Alfa Aesar) with Dithiothreitol (DTT) were added to each soluble sample for a final concentration of 1x. Sample buffer part 2 (250 mM Tris–HCl pH 6.8, 0.1% bromophenol blue, 40% glycerol) and DTT were added to each insoluble sample in 1:1, v:v ratio. Soluble samples were stored at − 20 °C or − 80 °C and insoluble samples were stored at room temperature for further analysis.

#### Co-immunoprecipitation

HT22 cells were transfected with pCDNA3, HA-CHCHD2^WT^, or HA-CHCHD2^T61I^ using FuGENE^®^ HD (Promega, E2311). Pierce^™^ Protein A/G Magnetic Beads (ThermoFisher, 88803) were blocked with 5% BSA in 1% CHAPS (ThermoFisher, 28300) in water overnight at 4 °C. 48 h post-transfection, cells were harvested using 1% CHAPS in water with protease and phosphatase inhibitors (GeneDEPOT, P3200-005 and P3100-010). Lysates were then pre-cleared with Pierce™ Protein A/G Magnetic Beads for 6 h with rotation at 4 °C. After pre-clearing, Cathepsin B (ProteinTech, 12216–1-AP) or Cathepsin L (ProteinTech, 27952–1-AP) antibodies (1:100, v:v) were added to pre-cleared lysates with blocked Pierce^™^ ProteinA/G Magnetic Beads and rotated at 4 °C overnight. Beads were washed with 1% CHAPS buffer five times with rotation, and bound proteins were eluted by boiling in 4× sample buffer (Alfa Aesar) with DTT, followed by Western blotting.

#### Cellular α-synuclein PFF clearance

Mouse HT22 cells were transfected with pCDNA3, HA-CHCHD2^WT^, or HA-CHCHD2^T61I^ constructs using FuGENE HD (Promega, E2311) and Opti-MEM. The following day, cells were treated with 1 μM of human αSyn PFFs (Abcam, ab218819) that had been sonicated with a water bath sonicator (QSonica, Q800R3) at 50% amplitude for 3 min at RT for 4 h. Cells were then washed twice with 1× PBS and complete media was replaced. Cells were fixed after 0 and 45 h with or without 1% triton (Bio-Rad, 1610407) and stained with a human-specific αSyn antibody (Enzo Life Sciences, ALX-804-258-L001) and CHCHD2. Successful uptake of αSyn PFFs was confirmed via Z-stack imaging. Mean intensities of αSyn were measured per cell and only transfected cells (as determined by CHCHD2 staining) were quantified for the D2^WT^ and D2^T61I^ conditions. Data were normalized as a percent of the vector control 0 h condition per experiment and then combined.

### Tissue harvesting

Tissue harvesting was performed as previously described [33]. Briefly, tissues were sonicated in RIPA buffer (50 mM Tris–HCl pH 7.4, 150 mM NaCl, 2 mM EDTA, 1% NP-40, 0.1% sodium dodecyl sulfate) until the sample was fully homogenized and centrifuged at 4 °C and 15,000 r.p.m. (Eppendorf 5424R) for 15 min. The supernatant was decanted into a new microcentrifuge tube; this is the RIPA-soluble fraction. The pellet was washed with 100 µL of RIPA then sonicated in sample buffer part 1 (10% sodium dodecyl sulfate, 250 mM Tris–HCl pH 6.8) until fully homogenized; this is the RIPA-insoluble sample. A BCA kit (BCA protein assay, Pierce, 23225) and a Biotek microplate reader were used to determine protein concentration. Soluble and insoluble samples were equalized to the same concentration. 4× sample buffer (Alfa Aesar) with Dithiothreitol (DTT) were added to each soluble sample for a final concentration of 1x. Sample buffer part 2 (250 mM Tris–HCl pH 6.8, 0.1% bromophenol blue, 40% glycerol) and DTT were added to each insoluble sample in 1:1, v:v ratio. Soluble samples were stored at − 20 °C or − 80 °C and insoluble samples were stored at room temperature for further analysis.

### Isolation of lysosomes

Lysosomes were isolated from mouse brains with the Minute^™^ Lysosome Isolation Kit (Invent Biotechnologies, LY-034) using the manufacturer’s protocol. Briefly, cortical tissues were homogenized in buffer A with inhibitors (GeneDEPOT, P3200-005 and P3100-010) with a Fisherbrand^™^ Bead Mill 24 Homogenizer, centrifuged at 1200xg for 10 min at 4 °C to remove cell debris, incubated on ice for 5 min, and spun down multiple times at increasing speeds to isolate nuclear and mitochondrial fractions. Buffer B with inhibitors was added, the samples incubated on ice for 30 min, and spun down at 11,000xg for 10 min at 4 °C to isolate lysosomes. Lysosomes were then harvested with RIPA plus inhibitors and spun down at 15,000 r.p.m (Eppendorf 5424R) for 15 min at 4 °C to separate the soluble and insoluble lysosomal fractions.

### Sarkosyl extraction of human cortex

Protein extraction from human cortex was performed as previously described [33, 74, 75]. Briefly, brain tissue was sonicated in cold extraction buffer (25 mM Tris–HCl, pH 7.4, 150 mM NaCl, 1 mM EDTA, 1 mM EGTA) with inhibitors, centrifuged at 80,000xg for 15 min at 4 °C, and the supernatants collected; this is the soluble fraction. A68 buffer (10 mM Tris–HCl, pH 7.4, 0.8 M NaCl, 10% Sucrose, 1 mM EGTA) with inhibitors was then added to the pellet, sonicated, and centrifuged at 400xg for 20 min at 4 °C. The supernatant was moved to a new tube, a 1% Sarkosyl solution was added, and incubated at room temperature for 1.5 h. Tubes were then centrifuged at 80,000xg for 30 min at room temperature. The supernatant was discarded, and the pellets were resuspended in 50 mM Tris–HCl pH 7.4; this is the insoluble fraction.

### Mitochondrial subfractionation

Mitochondrial subfractionation was performed as previously described [33, 56]. Briefly, mitochondria were isolated from fresh tissue using the ThermoFisher mitochondrial isolation kit for tissue (89801), washed and then resuspended in swelling buffer (10 mM KH_2_PO_4_, pH 7.4) for 20 min at 4 °C. An equal volume of shrinking buffer (10 mM HEPES, 10 mM MgCl_2_, 32% sucrose, 30% glycerol, pH 7.4) was added and resuspended for 15 min followed by centrifugation at 10,000xg for 15 min at 4 °C. The supernatant was ultracentrifuged at 100,000xg for 40 min at 4 °C to separate the outer membrane (OM) and intermembrane space (IMS) fractions, and the pellet was washed twice with washing buffer (250 mM sucrose, 1 mM EGTA, 10 mM HEPES, pH 7.4), resuspended in swelling buffer, sonicated and subjected to centrifugation at 12,000xg for 15 min at 4 °C. The supernatant was then ultracentrifuged at 100,000xg for 40 min at 4 °C to separate the IM and matrix fractions. All fractions were harvested with RIPA buffer with inhibitors, plus 4× sample buffer (Alfa Aesar) with Dithiothreitol (DTT) prior to Western blotting.

### Western blotting

Western blotting was performed as previously described [33]. Briefly, equalized samples were boiled at 100 °C for 10 min, or left unboiled if immunoblotting for CHCHD2, then subjected to SDS-PAGE electrophoresis. The proteins were transferred to 0.45 µm nitrocellulose membranes (GE Healthcare, 10600002), membranes were blocked with 5% skim milk in TBS-T for one hour at room temperature, and primary antibodies in TBS-T buffer were applied overnight at 4 °C with continuous shaking. HRP secondary antibodies (1:1000, v:v) diluted in blocking solution were applied overnight at 4 °C or for four hours at room temperature with continuous shaking, then blots were developed with ECL western blot reagents (Pierce, 34578) on a Fuji LAS-4000 imager (LAS-4000, Pittsburgh, PA, USA) or Cytiva Amersham ImageQuant^TM^800 (Cytiva, Marlborough, MA, USA). Image quantification was performed using ImageJ Software (National Institutes of Health, Bethesda, MD, USA). All measurements were normalized to their respective actin levels or Ponceau S for total protein staining. A list of all antibodies used can be found in Table 1.

### Immunohistochemistry

Immunohistochemistry was performed as previously described [33]. Briefly, PBS-perfused brains were post-fixed in 4% paraformaldehyde at 4 °C for 72 h, then replaced with 30% sucrose for cryoprotection. The tissues were sectioned (25 μm) coronally or horizontally (American Optical Microtome, AO-860 or Leica CM1860), washed with 0.2% Triton in TBS, boiled in 0.5 M citrate buffer pH 6.0 (ThermoScientific, J63950.AP) for five minutes for epitope retrieval, then blocked in 3% NGS and 0.2% Triton in PBS for one hour at room temperature. Tissues were then incubated with primary antibodies in blocking at 4 °C overnight with continuous shaking, washed with PBS, and secondary Alexa fluor antibodies (1:1000, v:v) in blocking solution were applied for 45 min at room temperature with continuous shaking. Tissues were washed with PBS for the final time, mounted on slides (Fisher Scientific, 12-544-7) and fully dried. Mounting solution (Invitrogen, 00-4958-02) was applied along with a glass coverslip (Fisher Scientific, 125485P) and left to dry for at least 12 h. Slides were imaged with the Keyence BZ-800 microscope (Keyence, Osaka, Japan) or ZEISS LSM880 confocal microscope (ZEISS, Jena, Germany). Signal intensities were quantified using ImageJ software (National Institutes of Health, Bethesda, MD, USA) and investigators were blind to genotypes during quantification. GFAP and Iba1 immunoreactivities were quantified by measuring the mean intensities of each region and subtracting background measurements taken between glia for each image. All IHC quantifications are normalized to wild-type littermates from the same staining experiment. A list of all antibodies used can be found in Table 1.

#### Quantification of TH-positive neurons

To quantify the number of dopaminergic neurons in the substantia nigra pars compacta (SNc), every four 25 μm thick coronal slices were collected from − 2 to − 4 mm from the bregma from 12-month-old WT and CHCHD2^WT^ brains. The tissues were immunostained for TH and DAPI (using the above IHC methods) and imaged with the Keyence BZ-X800 microscope (Keyence, Osaka, Japan). 20× Z-stack stitches were taken of the ventral midbrain with a pitch of 0.5 μm and a consistent Z-stack depth (11.5 μm) across all pieces. Intensities were also kept consistent across all images. Images were then turned into full-focus images and stitched together across the entire ventral midbrain region using Keyence BZ-X800 analysis software. A blinded investigator then used the Allen coronal mouse brain atlas (https://mouse.brain-map.org/static/atlas) and “The Mouse Brain in Stereotaxic Coordinates” by Keith B.K. Franklin and George Paxinos to determine the boundary between the VTA and SNc. Blinded investigators then used ImageJ software to count the number of TH-positive neurons in the SNc from 8 to 11 pieces per mouse ranging from − 2.8 and − 3.8 mm from the bregma. Cell counts per piece were then normalized as a percent of age-matched wild-type littermates prior to being combined.

### Silver staining

Silver staining was performed in free-floating sections using the FD NeuroSilver Kit II following the manufacturer protocol (FD NeuroTechnologies, Columbia, MD; PK301) as previously reported [11]. Images were acquired by a ZeissAxio Scan.Z1, keeping exposition time and light intensity constant. All the images were analyzed using ImageJ/Fiji version 2.14.0/1.54f software (NIH, Bethesda, MD). Color Deconvolution plugin was used for the silver staining analysis described by Ruifrok and Johnston [64].

### Cathepsin activity assays

Plate-based fluorometric cathepsin B, D, and L activity assays were purchased from Abcam (ab65300, ab65302, ab65306). Fresh forebrains were washed with ice-cold 1× PBS, minced on ice and separated equally into 3 tubes. Cathepsin B, D, and L lysis buffers were added to each respective tube, and samples were sonicated with a water bath sonicator (QSonica, Q800R3) at 60% amplitude until fully homogenized. Samples were spun down at 4 °C and 15,000 r.p.m. (Eppendorf 5424R) for 5 min and supernatants transferred to a new tube. A BCA kit (BCA protein assay, Pierce, 23225) and a Biotek microplate reader were used to determine protein concentration, and samples were equalized. Total protein concentrations were normalized to 2 μg/uL and 50 μL/well was loaded into black 96-well plates with clear bottoms. We also prepared background control wells with 50 μL of lysis buffer, negative control wells with cathepsin inhibitors, and positive control wells with cathepsin activator. A master mix of assay buffer and substrates was prepared per the manufacturer’s instruction, and 50 uL was added on top of all wells. Plates were then shaken at 37 °C protected from light for 2 h, then read with a BMG Labtech VANTAstar plate reader at Ex/Em 400/505 nm for cathepsins B and L activity, and Ex/Em 328/460 nm for cathepsin D activity. The background absorbance was subtracted from all values for each cathepsin activity assay.

### Electrophysiology

#### Hippocampal brain slice preparation

Acute hippocampal slice preparation was performed as previously described [33, 76]. Briefly, 10-month-old mice (C57BL/6 J or CHCHD2^WT^) were first decapitated. The brain was removed from the skull and put into ice-cold and oxygenated cutting solution (110 mM sucrose, 60 mM NaCl, 3 mM KCl, 28 mM NaHCO_3_, 1.25 mM NaH_2_PO_4_, 5 mM glucose, 0.6 mM ascorbate, 7 mM MgCl_2_, and 0.5 mM CaCl_2_). 400 μm parasagittal sections were generated in cutting solution using Leica VT1200. The hippocampus slice was dissected and transferred to room temperature cutting solution diluted 1:1 with artificial cerebrospinal fluid (ACSF) (125 mM NaCl, 2.5 mM KCl, 26 mM NaHCO_3_, 1.25 mM NaH_2_PO_4_, 25 mM glucose, 1 mM MgCl_2_, and 2 mM CaCl_2_), for 10 min. Then slices were incubated in ACSF with constant 95% O_2_/5% CO_2_ perfusion for 30 min before being loaded into the brain slice recording chamber (Scientific Systems Design Inc.).

#### Ex vivo electrophysiology recording

The long-term potentiation (LTP) recording was previously described [33, 76]. Slices were loaded into the recording chamber after a 30-min recovery. The recording chamber was held at 30° ± 0.5 °C with an ACSF flow rate of 1 mL/min. Field excitatory postsynaptic potential (fEPSP) was recorded from stratum radiatum in the hippocampal CA1 area via a glass electrode (1–4 MΩ) loaded with ACSF. A stimulating electrode made by the formvar-coated nichrome wire (A-M Systems) was positioned on the Schaffer collaterals arising from the CA3 region, which was used to deliver biphasic stimulus pulses (1–18 V, 100 μs duration). The electric stimulation, controlled by pClamp 11 software (Molecular Devices), was delivered via the Digidata 1550B interface (Molecular Devices) and a stimulus isolator (model 2200; A-M Systems). Signals were amplified using a differential amplifier (model 1800; A-M Systems), filtered at 1 kHz, and digitized at 10 kHz. The input–output relationship was determined by stimulating slices from 0 to 18 V at 0.5 V increments. A stimulus intensity, which would evoke ∼50% of the maximum fEPSP response, was determined from the input–output. This stimulus intensity was applied to the electrophysiological recording. Paired-pulse facilitation (PPF), which is a short-term plasticity, was induced with two sequential pulses and the interval was increased incrementally every 20 ms from 20 to 300 ms. A 20-min baseline fEPSP was recorded at 0.05 Hz as a control before the LTP evocation. LTP was induced by a theta burst stimulation (TBS), which consisted of five trains of four pulses at 200 Hz separated by 200 ms, repeated six times with an inter-train interval of 10 s. A 60-min fEPSP was recorded at 0.05 Hz following the TBS.

#### Statistical analysis

Data in bar graphs are shown as the mean ± SEM. The evoked fEPSP measured by the initial slope after the TBS was normalized to the averaged baseline slope of each hippocampal brain slice. For the PPF, the second fEPSP slope was normalized to the first fEPSP slope. For the Input–output experiment, the amplitude of fiber volley (FV) and the fEPSP slope were measured. FV^1^/_2_, which would be the fiber volley that evokes a ^1^/_2_ maximal fEPSP response, is a value that will normalize the fiber volley. The maximal fEPSP response will be used to normalize the fEPSP slope. A sigmoidal function (FV vs. fEPSP) for each slice and a predicted value from this curve was generated.

### Interactome screen

MTS-BirA, MTS-BirA-CHCHD2^WT^, and MTS-BirA-CHCHD2^T61I^ HT22 stable cells were cultured in 5 μg/mL of puromycin (ThermoFisher, A1113803) for selection. Each stable cell line was grown in triplicate (75cm^2^ flasks per condition) and treated with 50 μM of biotin (Sigma, B4639) for 12 h, washed twice with 1× PBS, and cells lysed with 1× RIPA (RPI, R26200) with 1 mM EDTA and 1 mM EGTA plus protease (GeneDEPOT, P3200-005), phosphatase (GeneDEPOT, P3100-010), and deacetylase inhibitors (APExBIO, K1017). Cells were incubated in 1× RIPA for 1 h at 4 °C for lysis, sonicated with a probe sonicator (QSonica Q125) at 65% amplitude on ice, and centrifuged at 15,000 r.p.m (Eppendorf 5424R) for 15 min at 4 °C. The supernatant was transferred to a new tube, and protein concentrations equalized. Streptavidin magnetic beads (Cytiva) were blocked in 4% BSA in 1× RIPA overnight on a spinning rotor at 4 °C and the blocking solution was washed off with 1× RIPA prior to addition to equalized cell lysates. Beads were incubated with lysates overnight at 4 °C on a spinning rotor, followed by magnetic bead separation with a magnet and four washes in 1× RIPA. A solution of 5% SDS, 100 mM ammonium bicarbonate, and 25 mM biotin was added to dry beads to remove bound biotinylated proteins and stored at − 80 °C prior to mass spectroscopy analysis.

### Proteomics

Proteomic analysis was performed as previously described [33]. Proteomics for interactome analysis were performed in triplicate per condition, and proteomics from mouse brains were performed in quadruplicate per condition. Equal amounts of protein (100 µg) were processed for LC–MS/MS using s-traps (Protifi) [25, 82]. Briefly, proteins were reduced with dithiothreitol (DTT), alkylated with iodoacetamide (IAA), acidified using phosphoric acid, and combined with s-trap loading buffer (90% MeOH, 100 mM TEAB). Proteins were loaded onto s-traps, washed with s-trap loading buffer, and finally digested with Trypsin/Lys-C (1:100, w:w; enzyme:protein) overnight at 37 ˚C. Peptides were eluted and dried with a vacuum concentrator. Peptides were resuspended in H_2_O/1% acetonitrile/0.1% formic acid for LC–MS/MS analysis. Peptides were separated using a 75 µm × 50 cm C18 reversed-phase-HPLC column (Thermo Scientific) on an Ultimate 3000 UHPLC (Thermo Scientific) using a 120-min gradient (2–32% ACN with 0.1% formic acid) and analyzed on a hybrid quadrupole-Orbitrap instrument (Q Exactive Plus, ThermoFisher Scientific). Full MS survey scans were acquired at 70,000 resolution. The top 10 most abundant ions were selected for MS/MS analysis. Raw data files are processed in MaxQuant (v 1.6.14.0, www.maxquant.org) and searched against the current Uniprot *Mus musculus* protein sequences database with the sequence for human CHCHD2 added. Search parameters included constant modification of cysteine by carbamidomethylation and the variable modifications, methionine oxidation and protein N-term acetylation. Proteins were identified using the filtering criteria of 1% protein and peptide false discovery rate. Protein intensity values were normalized using the MaxQuant LFQ function [14]. LFQ Intensity values were Log_2_-transformed for the creation of volcano plots. If a protein was detected in at least 3 out of 4 replicates in one experimental group and not detected in the other experimental group, we assigned an LFQ value of 1 to undetected replicates, representing below the threshold of detection. Statistically significant changes in protein abundance are determined using Welch’s t-test p-values.

### Qiagen ingenuity pathway analysis (IPA)

IPA was performed as previously described [11]. Briefly, proteins altered between experimental groups with unadjusted *P* < 0.05 (−Log_10_(*P*-value)>1.3) were included in IPA. For analysis of canonical pathways, the following parameters were selected: confidence = Experimentally Observed OR High (predicted); tissues/cell lines = all brain cell types and brain-related cell lines; molecule types = all. Activation Z-scores ≥2 or ≤−2 of canonical pathways are considered significantly activated or inactivated, respectively. The significance in overlap of analysis-ready proteins with the total number of proteins in the reference canonical or toxicity pathway is expressed by Benjamini–Hochberg adjusted −Log_10_(*P*-value).

### Transmission electron microscopy (TEM)

Investigators were blind to genotypes during the preparation and acquisition of TEM images. Mice were anesthetized and euthanized by transcardial perfusion with fixative solution (4% paraformaldehyde in 0.1 M HEPES, pH 7.0–7.3) at a flow rate of 25 mL/min for 5 min. The brains were carefully removed, and the cortex was sliced into thin slices (3 mm × 3 mm × 2 mm) with a brain matrix. Tissues were fixed with triple aldehyde-DMSO [22] in 0.1 M HEPES buffer, pH 7.4 for 2 h at room temperature. The specimens were thoroughly rinsed in 0.1 M phosphate buffer, pH 7.4, and then post-fixed for 2 h in an unbuffered 1:1 mixture of 2% osmium tetroxide and 3% potassium ferricyanide. After rinsing with distilled water, specimens were soaked overnight in an acidified solution of 0.25% uranyl acetate. After another rinse in distilled water, specimens were dehydrated in ascending concentrations of ethanol, passed through propylene oxide, and embedded in EMbed 812 resin mixture (Electron Microscopy Sciences, PA). Thin Sects. (70 nm) were cut on an RMC MT6000-XL ultramicrotome and mounted on Gilder square 300 mesh nickel grids (Electron Microscopy Sciences, PA). Thin sections were sequentially stained with acidified uranyl acetate followed by a modification of Sato’s triple lead stain [26]. These were coated on a Denton DV-401 carbon coater (Denton Vacuum LLC, NJ), and examined in a FEI Tecnai Spirit (T12) with a Gatan US4000 4 k x 4 k CCD.

TEM images were quantified by counting the total number of mitochondria and lysosomes per cell and assigning a qualitative color to each organelle. Green and red mitochondria were characterized by dense cristae and an elongated shape, or less dense cristae and a rounded shape, respectively. Less electron dense lysosomes with few lipofuscin puncta and a rounded morphology were assigned as green, and more electron dense lysosomes with a moderate amount of lipofuscin and up to 1 lipid droplet were assigned as yellow. Red lysosomes were characterized as very electron dense with many lipofuscin puncta, an irregular shape, and multiple lipid droplets. Investigators were blind to genotypes during quantification.

### Human brain *Chchd2* expression

We utilized human brain microarray data from the study by Lu et al. (GDS5204) [45]. This dataset comprises a total of 41 samples from the frontal cortex, with subjects' ages ranging from 24 to 106 years. The samples were categorized into four age groups: 12 young individuals (< 40 years), 9 middle-aged individuals (40–70 years), 16 normal-aged individuals (70–94 years), and 4 extremely aged individuals (95–106 years). Using log_2_ transformed count data and age, we computed 2-tailed Pearson’s correlation coefficients to evaluate the relationship between *Chchd2* gene expression and age.

### Statistical analysis

All graphs were created and analyzed with GraphPad Prism 10 software (GraphPad Software, San Diego, CA, USA) using student’s *t*-test, one-way analysis of variance (ANOVA) followed by Tukey post hoc test, two-way ANOVA followed by Dunnett’s or Sidak post hoc test, Fisher’s exact test, χ^2^ test, or Pearson’s correlation analysis. Differences were deemed significant when *P* < 0.05. Quantified graphs are expressed as mean ± S.E.M.

**Table 1:** Antibody information

Target	Host	Supplier	Catalog Number	WB Dilution	IF/PLA/IP Dilution
Human alpha synuclein	Rat Monoclonal	Enzo life sciences	ALX-804-258-L001	1:100	IF 1:50
β-Actin	Mouse Monoclonal	ProteinTech	66009–1-Ig	1:5000	–
BirA	Mouse Monoclonal	Novus biologicals	5B11c3-3	1:1000	IF 1:100
Cathepsin B	Rabbit Monoclonal	Cell signaling	31718	1:1000	IF/PLA 1:200
Cathepsin B	Rabbit Polyclonal	ProteinTech	12216–1-AP	1:1000	IP 1:100
Cathepsin D	Rabbit Polyclonal	ProteinTech	55021–1-AP	1:1000	–
Cathepsin L	Rabbit Polyclonal	ProteinTech	27952–1-AP	1:1000	IF/PLA 1:200 IP 1:100
CHCHD2	Mouse Monoclonal	ProteinTech	66302–1-Ig	–	IF 1:200
CHCHD2	Rabbit Polyclonal	ProteinTech	19424–1-AP	1:1000	IF 1:200
CHCHD4	Mouse Monoclonal	Santa Cruz	sc-365137	1:1000	–
CLPP	Rabbit Polyclonal	Novus biologicals	NBP1-89557	1:1000	–
Galectin-3	Mouse Monoclonal	BioLegend	125402	–	IF 1:100
GAPDH	Mouse Monoclonal	ProteinTech	HRP-60004	1:2000	–
GFAP	Rat Monoclonal	Invitrogen	13–0300	–	IF 1:500
HA Tag	Mouse Monoclonal	Cell signaling	2367S	1:1000	IF/PLA 1:100
HEXA	Mouse Monoclonal	ProteinTech	60337–1-Ig	1:1000	–
Iba1	Rabbit Polyclonal	Fujifilm	09–19741	–	IF 1:500
LAMP1	Rabbit Monoclonal	Cell signaling	9091S	1:1000	–
LAMP2	Mouse Monoclonal	ProteinTech	66301–1-Ig	1:1000	–
M2 (Flag)	Mouse Monoclonal	Sigma-Aldrich	F1804	1:1000	IF 1:200
Mitofilin	Mouse Monoclonal	Santa Cruz	sc-390707	1:1000	IF 1:200
Synaptophysin	Rabbit Monoclonal	Abcam	ab32127	–	IF 1:500
TH	Sheep Polyclonal	Invitrogen	PA1-4679	–	IF 1:200
TH	Mouse Monoclonal	Invitrogen	MA1-24654	–	IF 1:200
Tom70	Mouse Monoclonal	Santa Cruz	sc-390545	1:1000	–

## Results

### CHCHD2 expression declines with age in the human brain

Recent studies indicate that *Chchd2* mRNA and D2 protein levels are significantly reduced in erythrocytes and the substantia nigra (SN) of PD patients, with diminished erythrocyte *Chchd2* mRNA serving as a prognostic indicator for early PD (> 80% sensitivity and specificity) [41]. Notably, similar reductions in *Chchd2* mRNA and D2 protein occur in erythrocytes and brains of αSyn^A53T^ transgenic mice [41]. Our prior work further demonstrated decreased cortical D2 levels in cortical LBD patients compared to controls [33].

Given that aging is the primary risk factor for neurodegenerative diseases, including LBDs [62], we investigated whether D2 levels decline with age in cognitively normal human brains. Analysis of a published cortical microarray dataset [45] revealed a significant age-dependent reduction in *Chchd2* gene expression between 20 to 100 years of age (Fig. 1a). This decline was observed in both sexes, with females exhibiting a steeper regression slope and stronger statistical significance (Fig. 1b) than males (Fig. 1c). These findings align with aged D2-deficient mice, which show dopaminergic neuron loss, mitochondrial fragmentation, and SQSTM1/p62 inclusions [66], suggesting that age-related D2 reduction may heighten LBD susceptibility by impairing mitochondrial-lysosomal homeostasis.

### CHCHD2 mitochondrial localization and intact synaptic plasticity in D2^WT^ mice

Given the deleterious effects of D2 loss in the aging brain [66], we hypothesized that augmenting D2 levels could confer neuroprotection. To address this, we generated D2^WT^ transgenic mice using the neuronal mouse prion protein (*mPrP*) promoter strategy we previously employed for the D2^T61I^ mice [33], enabling brain-specific expression of human Flag-D2 (Fig. 1d, and Fig. S1a and b).

Whole-brain lysates from D2^WT^ and WT littermate P0 pups clearly indicate the expression of exogenous human Flag-D2 (Fig. 1e), and qRT-PCR analysis revealed a ~2.3-fold increase in *Chchd2* transcripts (mouse + human) in 12-month-old D2^WT^ brains compared to WT controls, identical to the level observed in D2^T61I^ mice (Fig. 1f). However, Western blot analysis of total protein lysates from 5-month-old WT, D2^WT^, and D2^T61I^ brains revealed ~50% less Flag-D2 protein in D2^T61I^ compared to D2^WT^ in multiple CNS regions (fig. S1c–g), possibly reflecting differences in D2 stability. D2^WT^ pups exhibited no phenotypic abnormalities or differences in body weight compared to WT littermates (Fig. 1g and h). By 12-months of age, brain and spinal cord morphology remained normal in D2^WT^ mice (Fig. S1h), with horizontal brain sections (Fig. 1i, and Fig. S1i) from 12-month-old D2^WT^ and RIPA-soluble lysates (Fig. 1j) from 18-month-old D2^WT^ mice confirming widespread transgenic Flag-D2 expression across the cortex, midbrain, hippocampus, and spinal cord.

To validate mitochondrial localization, we performed sub-fractionation of isolated mitochondria into outer membrane (OM), intermembrane space (IMS), inner membrane (IM), and matrix (MX). Flag-D2^WT^ localized predominantly to the IMS together with endogenous D2 (Fig. 1k), mirroring the distribution of Flag-D2^T61I^ [33]. Hippocampal synaptic integrity, assessed via synaptophysin intensity, was unaltered in 12-month-old D2^WT^ mice (Fig. 1l and m), in contrast to the significant reduction in D2^T61I^ mice we previously reported [33]. Electrophysiological recordings further demonstrated normal input–output (I-O) curves, paired-pulse facilitation (PPF), and long-term potentiation (LTP) in the hippocampal CA3-CA1 Schaffer collateral pathway at 10 months of age (Fig. 1n to p), contrasting with the impaired LTP observed in age-matched D2^T61I^ mice [33]. These findings confirm that D2^WT^ mice exhibit proper mitochondrial targeting, intact synaptic plasticity, and no overt phenotypic deficits, establishing these mice as a robust tool for studying D2-mediated neuroprotection.

### CHCHD2^T61I^, but not CHCHD2^WT^ mice, exhibit cognitive deficits

To evaluate cognitive function, we performed novel object recognition (NOR) testing in 18-month-old D2^WT^, D2^T61I^, and WT littermate mice. The NOR test assesses innate exploratory behavior without aversive stimuli, enabling direct evaluation of recognition memory [46]. Mice underwent a standard 3 day NOR protocol: habituation (day 1), exposure to two identical objects (day 2), and replacement of one object with a novel counterpart (day 3) (Fig. 1q). Recognition memory was quantified using the discrimination index (novel object exploration time / total time exploring both objects).

Aged WT mice displayed a robust discrimination index of ~0.7 (Fig. 1r), indicating intact recognition memory. Likewise, aged D2^WT^ mice did not show any difference in discrimination index compared to WT mice (Fig. 1r), indicating normal recognition memory in vivo. By contrast, aged D2^T61I^ mice showed a significantly lower discrimination index of <0.6 compared to WT and D2^WT^ mice (Fig. 1r), approaching chance levels and thereby indicating profound recognition memory impairment. This differs from the normal performance of 12-month-old D2^T61I^ mice in contextual fear conditioning [33], suggesting age-related deterioration by 18 months. Open field testing revealed no differences in locomotor activity or anxiety-like behavior across genotypes (Fig. S1j to q), confirming that memory deficits in D2^T61I^ mice could not be attributed to any differences in motor or exploratory behavior.

### CHCHD2^WT^ mitigates age-related neuroinflammation, dopaminergic neuron loss, and insoluble proteinopathy

**Fig. 2:**
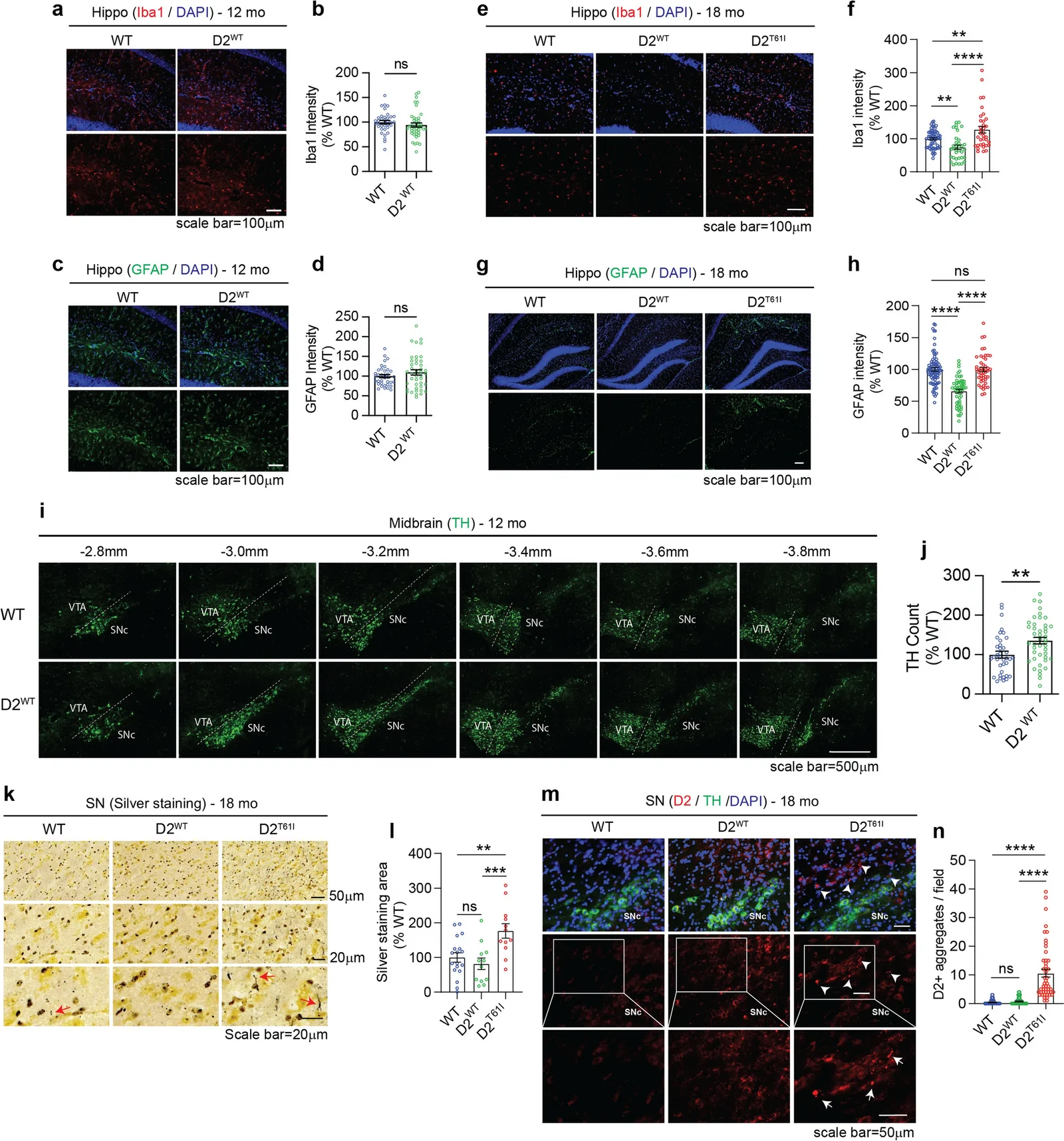
CHCHD2^WT^ protects against age-related neuroinflammation, dopaminergic neuron loss, and insoluble proteinopathy. **a** Representative images of hippocampal tissues from 12-month-old WT and CHCHD2^WT^ mice immunostained for Iba1 (red) and DAPI (blue). **b** Quantitative analysis of hippocampal Iba1 intensity (*t* test, *ns* not significant, n = 36–42 images per condition from 6 WT and 8 CHCHD2^WT^). **c** Representative images of hippocampal tissues from 12-month-old WT and CHCHD2^WT^ mice immunostained for GFAP (green) and DAPI (blue). **d** Quantitative analysis of hippocampal GFAP intensity (*t* test, *ns* not significant, n = 36–42 images per condition from 6 WT and 8 CHCHD2^WT^). **e** Representative images of hippocampal tissues from 18-month-old WT, CHCHD2^WT^, and CHCHD2^T61I^ mice immunostained for Iba1 (red) and DAPI (blue). **f** Quantitative analysis of hippocampal Iba1 intensity (one-way ANOVA; post hoc Tukey, **P < 0.005, ****P < 0.0001, *n* = 35–61 images per condition from 8 WT, 4 CHCHD2^WT^, and 5 CHCHD2^T61I^). **g** Representative images of hippocampus from 18-month-old WT, CHCHD2^WT^, and CHCHD2^T61I^ mice immunostained for GFAP (green) and DAPI (blue). **h** Quantitative analysis of hippocampal GFAP intensity (one-way ANOVA; post hoc Tukey, ****P < 0.0001, *ns* not significant, n = 47–84 images per condition from 8 WT, 4 CHCHD2^WT^, and 5 CHCHD2^T61I^). **i** Representative full-focus images of ventral midbrain slices from 12-month-old CHCHD2^WT^ Tg and WT littermates immunostained for TH (green) to label dopaminergic neurons. The top numbers correspond to the approximate distance from the bregma. *VTA* ventral tegmental area, *SNc* substantia nigra pars compacta. **j** Quantitative analysis of the number of TH+ dopaminergic neurons in the SNc (*t* test, **P < 0.005, *n* = 8–11 slices per condition from 4 WT and 5 CHCHD2^WT^). **k** Representative images of silver-stained substantia nigra (*SN*) from 18-month-old WT, CHCHD2^WT^ and CHCHD2^T61I^ mice. **l** Quantitative analysis of the % area of silver staining in the SN (one-way ANOVA; post hoc Tukey, **P < 0.005, ***P < 0.001, *ns* not significant, n = 12–18 images per condition from 6 WT, 4 CHCHD2^WT^, and 7 CHCHD2^T61I^). **m** Representative images of midbrain sections from 18-month-old WT, CHCHD2^WT^, and CHCHD2^T61I^ mice immunostained for CHCHD2 (red), TH (green), and DAPI (blue). White arrows indicate D2+ aggregates. **n** Quantitative analysis of CHCHD2-positive aggregates in the midbrain (one-way ANOVA; post hoc Tukey, ****P < 0.0001, *ns* not significant, n = 45–72 images per condition from 4 mice/genotype)

Neuroinflammation is a hallmark of age-related neurodegenerative diseases, and age-associated increases in astrocytic and microglial reactivity, as reflected by elevated GFAP and Iba1 immunoreactivity, are well established [10, 15, 35, 68]. We previously reported that 12-month-old D2^T61I^ mice exhibit elevated neuroinflammation relative to WT littermates, as quantified by Iba1 and GFAP immunoreactivity [33]. In contrast, we observed here that 12-month-old D2^WT^ mice display no changes in Iba1 (Fig. 2a and b, and Fig. S2a) or GFAP (Fig. 2c and d, and Fig. S2b). Strikingly, however, 18-month-old D2^WT^ mice showed significant reductions in both Iba1 (Fig. 2e and f, and Fig. S2c to e) and GFAP (Fig. 2g and h, and Fig. S2f) immunoreactivity compared to age-matched D2^T61I^ and WT littermates, suggesting that D2^WT^ expression attenuates aging-associated neuroinflammation. While Iba1 intensity increased in 18-month-old D2^T61I^ mice compared to WT littermates (Fig. 2e and f, and Fig. S2c and d), GFAP levels remained unchanged (Fig. 2g and h), indicating progressive microgliosis but stable astrogliosis in D2^T61I^ mice with aging.

Our prior work revealed a ~30% loss of tyrosine hydroxylase-positive (TH +) dopaminergic neurons in the SN of 12-month-old D2^T61I^ mice compared to WT littermates [33]. In contrast, 12-month-old D2^WT^ mice exhibited a significant increase in the number of TH+ dopaminergic neurons within the SN compared to WT littermates (Fig. 2i and j, and Fig. S2g). Consistent with this finding, D2 overexpression was previously shown to attenuate MPTP-induced loss of TH-positive neurons in both the SN and striatum [73]. These results imply that these cells are highly sensitive to D2 expression and that D2^WT^ protects dopaminergic neurons from age-related loss.

In assessing axonal integrity, silver staining in the SN of 18-month-old WT, D2^WT^, and D2^T61I^ mice revealed a near 2-fold increase in degenerating (silver-positive) axons in D2^T61I^ mice versus WT and D2^WT^ mice (Fig. 2k and l, red arrows). Although D2^WT^ mice trended toward reduced silver staining compared to WT mice, this difference lacked statistical significance (Fig. 2k and l). Immunostaining for D2 in 18-month-old mice revealed prominent aggregate-like D2 inclusions in the midbrain of D2^T61I^ mice compared to D2^WT^ and WT mice (Fig. 2m and n, white arrows, and Fig. S2h), indicating that mutant D2 pathologically accumulates in the aging brain.

**Fig. 3:**
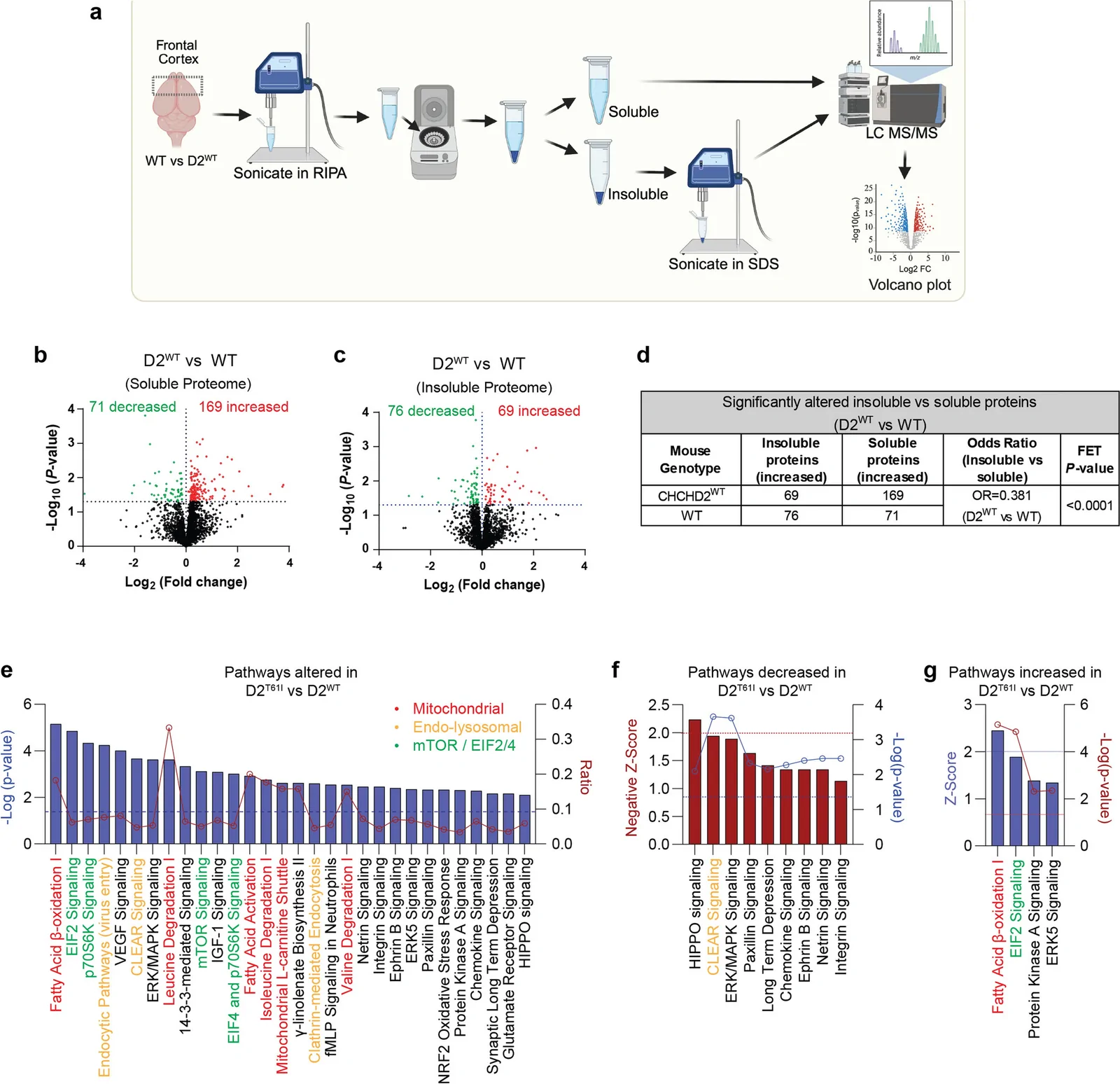
Disruption of the mitochondria-lysosome axis in brains of CHCHD2^T61I^ mice. **a** Representative schematic of the experimental flow for unbiased proteomic analysis of RIPA soluble and insoluble cortical fractions. Created with BioRender.com. **b** Volcano plot of the RIPA soluble proteome and **c** RIPA-insoluble proteome of 12-month-old CHCHD2^WT^ and WT littermate cortex (n = 4mice/genotype). **d** Contingency table listing the number of significantly altered RIPA insoluble and soluble proteins in 12-month-old CHCHD2^WT^ mice compared to WT littermates. *FET* Fisher’s exact test. **e** Qiagen^®^ Ingenuity^®^ pathway analysis (*IPA*) results of the top 30 canonical pathways altered in 12-month-old CHCHD2^T61I^ versus CHCHD2^WT^ cortex plotted by −Log(p-value) and ratio. Dotted blue line (≥ 1.3) = significant adjusted −Log(p-value) for overlapping genes in each pathway. **f** IPA results of pathways significantly decreased in 12-month-old CHCHD2^T61I^ compared to CHCHD2^WT^ cortex plotted by negative Z-score and -Log(p-value) among the top 30 pathways. Dotted red line (≥ 2) = significant pathway suppression Z-score. Dotted blue line (≥ 1.3) = significant adjusted −Log(p-value) for overlapping genes in each pathway. **g** IPA results of pathways significantly increased in 12-month-old CHCHD2^T61I^ compared to CHCHD2^WT^ cortex plotted by positive Z-score and −Log(p-value) among the top 30 pathways. Dotted blue line (≥ 2) = significant pathway activation Z-score. Dotted red line (≥ 1.3) = significant adjusted −Log(p-value) for overlapping genes in each pathway

To explore the proteostatic effects of D2 in aging, we analyzed the RIPA-soluble and RIPA-insoluble cortical brain proteomes of 12-month-old D2^WT^ and WT mice via LC-MS/MS (Fig. 3a). Among 240 significantly altered soluble proteins, 169 were elevated in D2^WT^ vs WT brains (Fig. 3b). Conversely, the majority (76/145) of significantly altered insoluble proteins were decreased in CHCHD2^WT^ vs WT brains (Fig. 3c). Fisher’s exact test (FET) tabulating increased insoluble vs soluble proteins in D2^WT^ vs WT brains yielded an odds ratio of 0.381 with *P* < 0.0001 (Fig. 3d), demonstrating that D2^WT^ significantly decreases the ratio of insoluble to soluble proteins, contrasting sharply with our earlier findings in 10-month-old D2^T61I^ brains, which showed a marked increase in insoluble proteins (odds ratio = 23.4 with *P* < 0.0001) (Fig. S3a) [33]. These data support a protective role for D2^WT^ in maintaining proteostatic balance in the aged brain, in stark contrast to the aggregation-prone phenotype of D2^T61I^ [33] and age-associated accumulation of SQSTM1/p62 inclusions in D2 knockout mice [66].

### CHCHD2^T61I^ disrupts the mitochondria-lysosome axis in the brain

To identify pathway-specific alterations in 12-month-old D2^WT^ versus D2^T61I^ mice, we performed LC-MS/MS-based proteomic profiling of RIPA-soluble frontal cortex extracts. Ingenuity Pathway Analysis^®^ (IPA) of significantly altered proteins (Table S1) revealed broad dysregulation across three key functional themes within the top 30 canonical pathways that were significantly altered (−Log_10_(*P*)>1.3) (Fig. 3e, and Table S2). First, mitochondrial pathways related to fatty acid β-oxidation, fatty acid activation, leucine/isoleucine/valine degradation, and the L-carnitine shuttle were highly significantly altered (Fig. 3e, red, and Table S2), consistent with the primary localization of D2 to mitochondria. Second, the ‘Coordinated Lysosomal Expression And Regulation’ (CLEAR) signaling pathway and endocytic pathways were among the top significantly altered (Fig. 3e, orange, and Table S2), indicating stark differences in lysosome-related endocytic and degradation pathways. Third, integrated stress response and mTOR-related pathways were significantly altered, including EIF2, EIF4, p70S6K, and mTOR signaling pathways (Fig. 3e, green, and Table S2). Among these pathways with computable Z-scores, pathway activation states inferred from Z-scores further demonstrated suppression of CLEAR signaling (Z = − 1.9) (Fig. 3f, orange, and Table S2) and activation of fatty acid β-oxidation (Z = 2.3) (Fig. 3g, red, and Table S2) and EIF2 signaling (Z = 1.9) (Fig. 3g, green, and Table S2).

While mitochondrial roles for D2^WT^ and D2^T61I^ are well established, their impact on the endo-lysosomal system (i.e. CLEAR) and mTOR signaling pathways have not been previously noted. We therefore systematically analyzed lysosomal and mTOR-associated proteins. D2^T61I^ brains exhibited pronounced reductions in the lysosomal hydrolases cathepsin B (CTSB), cathepsin L (CTSL), and hexosaminidase A (HEXA) (Fig. S3b), and we confirmed the corresponding change in CTSL protein by Western blotting (Fig. S3c and d). Furthermore, LAMTOR3 and Rab39A, components of the mTOR pathway, were undetectable in D2^T61I^ brains but readily detectable in D2^WT^ from the proteomics dataset (Fig. S3b). No lysosomal proteins were upregulated in D2^T61I^ vs D2^WT^ brains, suggesting a unidirectional effect in lysosomal homeostasis. Collectively, these findings demonstrate that D2^T61I^ disrupts the mitochondria-lysosome axis, dysregulating mTOR signaling and integrated stress responses. This lysosomal dysfunction may mechanistically explain the exacerbated proteinopathy we previously observed in D2^T61I^ brains [33].

### CHCHD2^T61I^ preferentially binds and inhibits cathepsin B and L lysosomal proteases

**Fig. 4:**
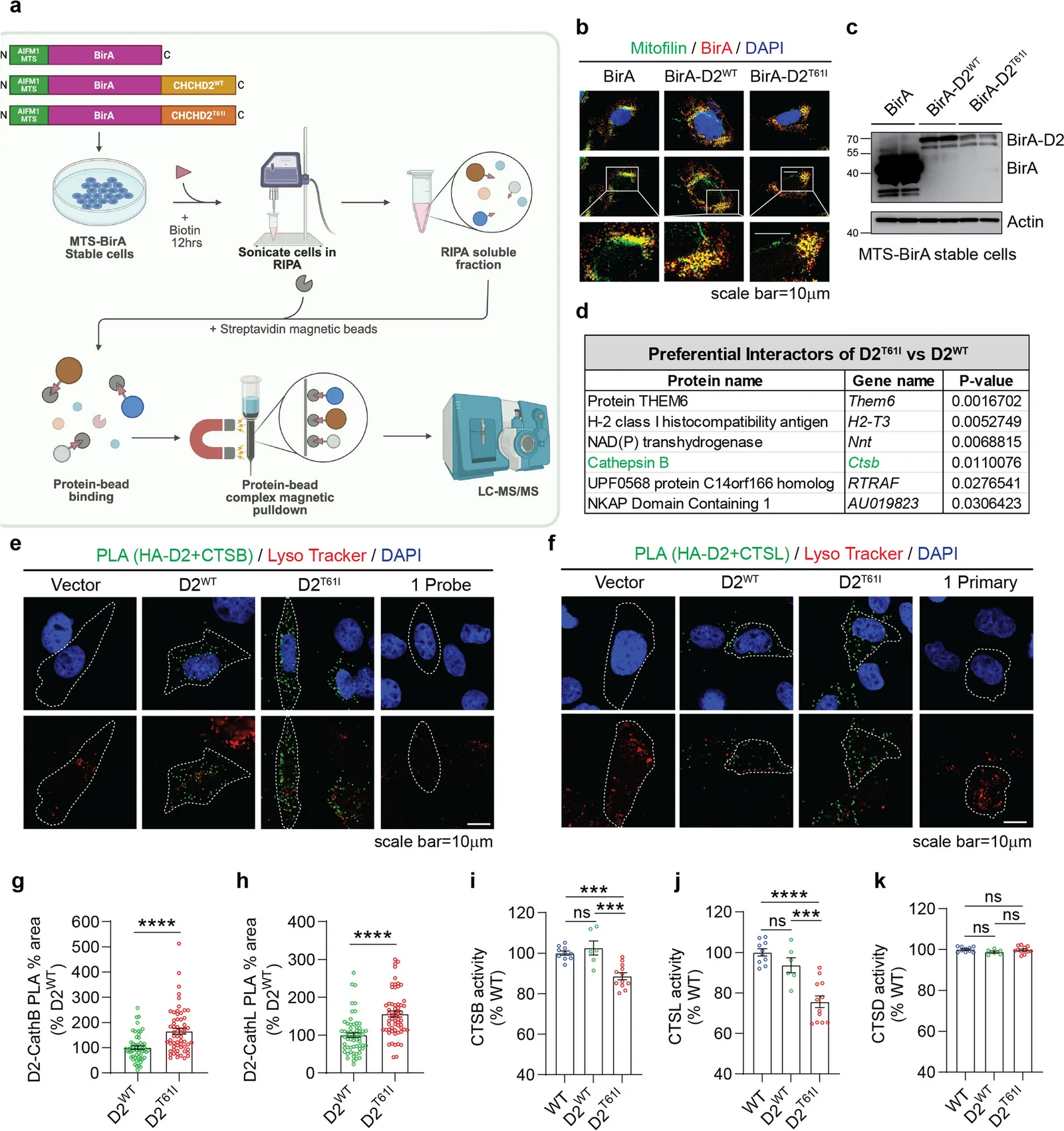
CHCHD2^T61I^ preferentially interacts with cathepsins B and L, reducing their activities. **a** Schematic of the unbiased interactome screen for MTS-BirA, MTS-BirA-CHCHD2^WT^ and MTS-BirA-CHCHD2^T61I^. Created with BioRender.com. **b** Representative images of MTS-BirA HT22 stable cell variants immunostained for BirA (red), mitochondrial mitofilin (green), and DAPI (blue). **c** Representative immunoblots of RIPA-soluble lysates from MTS-BirA HT22 stable cell variants for BirA and actin. **d** List of preferential interactors of CHCHD2^T61I^ compared to CHCHD2^WT^ from the interactome screen (n = 3/condition). **e** Representative images of PLA (HA-CHCHD2/CTSB interaction, green) and **f** PLA (HA-CHCHD2/CTSL interaction, red) in HT22 cells transfected with pcDNA3 vector, HA-CHCHD2^WT^, or HA-CHCHD2^T61I^ and treated with LysoTracker (red) to label healthy lysosomes. **g** Quantitative analysis of PLA (HA-CHCHD2/CTSB) puncta % area (*t* test, ****P < 0.0001, n = 6 wells/condition from 3 experiments) and **h** PLA (HA-CHCHD2/CTSL) PLA puncta % area (*t* test, ****P < 0.0001, n = 6 wells/condition from 3 experiments). **i**–**k** Quantitative analysis of cathepsin activities in forebrains of 10-month-old WT, CHCHD2^WT^, and CHCHD2^T61I^ mice (one-way ANOVA; post hoc Tukey, ***P < 0.001, ****P < 0.0001, *ns* not significant, n = 2 replicates/mouse from 6 WT, 5 CHCHD2^WT^, and 6 CHCHD2^T61I^)

To investigate differential protein interactions between D2^T61I^ and D2^WT^, we devised an unbiased screen to identify differentially interacting proteins using BirA-based proximity biotin labeling (BioID) (Fig. 4a). A BioID construct containing the N-terminal mitochondrial-targeting sequence (MTS) of AIFM1 fused to BirA, which targets fusion proteins to the IMS [3], was employed to target D2 to the mitochondrial IMS. D2^T61I^ or D2^WT^ were fused to the C-terminus of MTS-BirA, generating MTS-BirA-D2 constructs, which were confirmed by transient transfection (Fig. S4a). Lentiviral delivery of these constructs into HT22 mouse hippocampal neuroblastoma cells and puromycin selection produced stable cell lines, with mitochondrial localization confirmed by immunofluorescence via colocalization with the mitochondrial inner membrane protein mitofilin (Fig. 4b, and Fig. S4b), and expression confirmed via Western blotting (Fig. 4c).

For interactome profiling, biotin-treated cells were lysed with RIPA buffer and biotinylated proteins were isolated via streptavidin pulldown for LC-MS/MS analysis (Fig. 4a). Despite the lower expression of MTS-BirA-D2^T61I^ compared to MTS-BirA-D2^WT^ or MTS-BirA controls (Fig. 4c), we identified six proteins enriched in D2^T61I^ pulldowns in three replicates, including cathepsin B (Fig. 4d). Given our earlier proteomic findings of reduced CTSB and CTSL in D2^T61I^ brains (Fig. S3), we prioritized the validation of these interactions. Proximity ligation assays (PLAs) in HT22 cells expressing HA-D2^T61I^ or HA-D2^WT^ revealed significantly stronger interactions between D2^T61I^ and endogenous CTSB (~ 1.5–2-fold increase in PLA puncta area, Fig. 4e and g, and Fig. S4c and e) and CTSL (Fig. 4f and h, and Fig. S4d and f), compared to D2^WT^. Labeling lysosomes with LysoTracker, which labels compartments with a low pH, revealed a lack of colocalization between D2-CTSL complexes and healthy lysosomes (Fig. 4e and f and Fig. S4g to j). Co-immunoprecipitation also showed increased interactions of CTSB and CTSL with HA-D2^T61I^ (Fig. S4k and l). These results confirm the preferential binding of D2^T61I^ to lysosomal proteases CTSB and CTSL, but not in healthy low-pH lysosomal compartments.

To assess functional consequences, we performed CTSB, CTSL, and cathepsin D (CTSD) activity assays from 10-month-old WT, D2^WT^, and D2^T61I^ forebrains. D2^T61I^ brains exhibited significantly reduced CTSB and CTSL activities compared to WT and D2^WT^ (Fig. 4i and j), while CTSD activity remained unchanged (Fig. 4k), indicating the selectivity of D2^T61I^ to CTSB and CTSL.

### LBD brains and CHCHD2^T61I^ transfected cells exhibit reduced cathepsin B and L levels

**Fig. 5:**
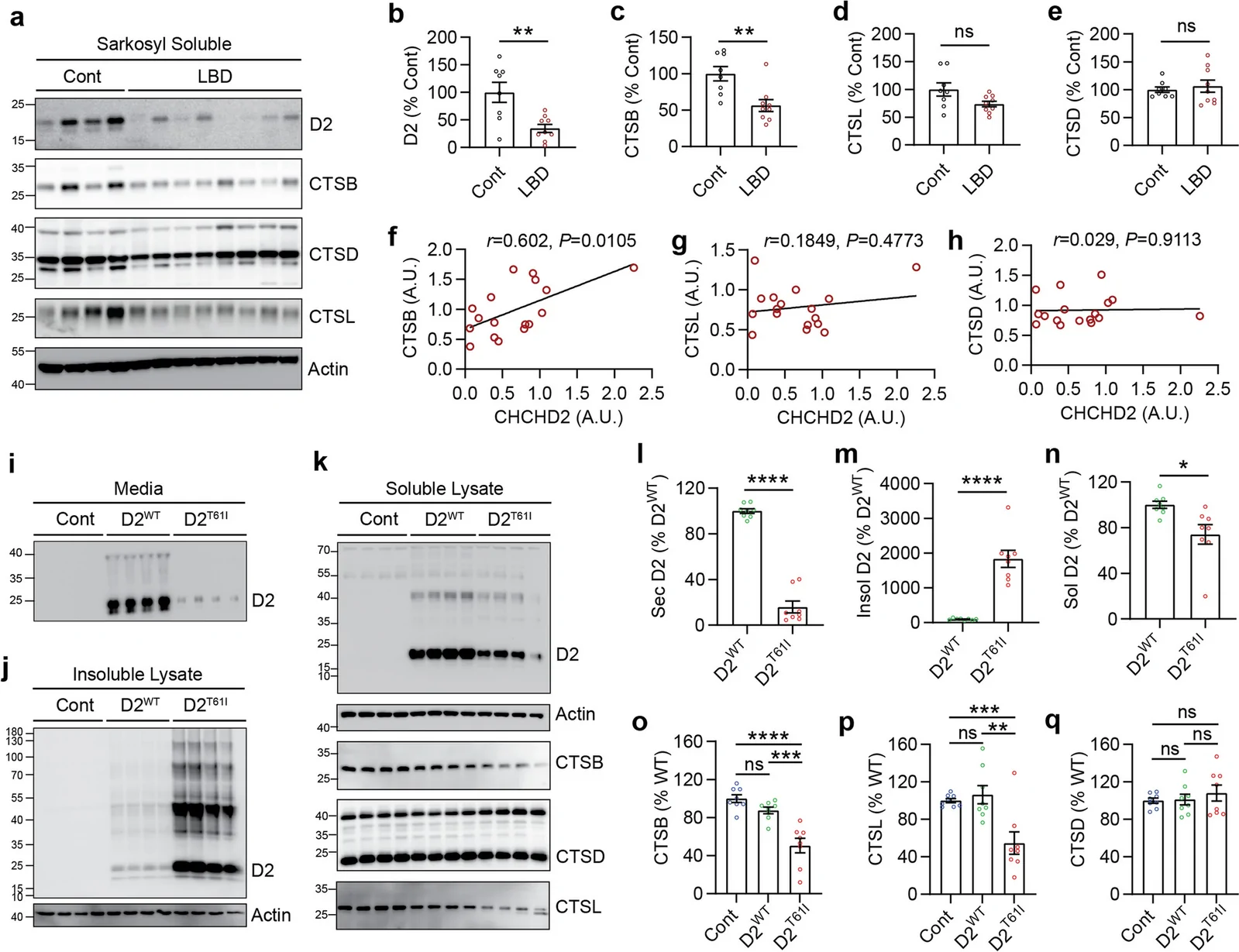
LBD brains and CHCHD2^T61I^ transfected cells exhibit reduced cathepsin B and L. **a** Representative immunoblots of cortical Sarkosyl soluble lysates from human control and LBD patients probed for CHCHD2 (D2), CTSB, CTSD, CTSL and actin. **b**–**e** Quantitative analysis of Sarkosyl soluble CHCHD2 (D2), CTSB, CTSL, and CTSD levels (*t* test, **P < 0.005, *ns* not significant, *n* = 8 control and 9 LBD patients from 2 experiments). Correlations of Sarkosyl soluble CHCHD2 with **f** CTSB (Pearson’s correlation, r = 0.602, p = 0.0105, n = 17), **g** CTSL (Pearson’s correlation, r = 0.1849, p = 0.4773, n = 17), and **h** CTSD (Pearson’s correlation, r = 0.029, p = 0.9113, n = 17) in human controls and LBD cortex. Representative immunoblots of **i** media, **j** RIPA soluble, and **k** RIPA insoluble lysates from HT22 cells transfected with pcDNA3 vector, HA-CHCHD2^WT^, or HA-CHCHD2^T61I^, probed for CHCHD2, CTSB, CTSD, CTSL and actin. Quantitative analysis of **l** secreted D2, **m** insoluble D2, and **n** soluble CHCHD2 (*t* test, *P < 0.05, ****P < 0.0001, n = 8 wells/condition from 2 experiments). **o–q** Quantitative analysis of RIPA-soluble cathepsin levels (one-way ANOVA; post hoc Tukey, **P < 0.005, ***P < 0.001, ****P < 0.0001, *ns* not significant, n = 8 wells/condition from 2 experiments)

To assess lysosomal protease alterations in LBDs, we performed Sarkosyl protein extraction from the frontal cortex of 9 cortical LBD patients and 8 age-matched controls (Fig. S5). Consistent with our previous report [33], soluble D2 levels were significantly reduced by ~65% in cortical LBD patients compared to controls (Fig. 5a and b). Similarly, CTSB levels decreased by ~45% in cortical LBD patients compared to controls (Fig. 5a and c), while CTSL showed a ~25% reduction that did not reach statistical significance (Fig. 5a and d). CTSD levels remained unchanged (Fig. 5a and e). Pearson’s correlation analysis revealed a significant positive correlation between D2 and CTSB levels (Fig. 5f) among LBD and control brains, but not for CTSL (Fig. 5g) or CTSD (Fig. 5h), suggesting a selective interplay between D2 and CTSB during LBD pathogenesis.

In considering how D2^T61I^ disrupts the mitochondria-lysosome axis, we hypothesized impaired lysosomal trafficking of mitochondrial D2. Mitochondrial quality control typically involves bulk mitophagy [47, 80] or mitochondria-derived vesicles, with the latter either routed to lysosomes or secreted as small extracellular vesicles [50, 61, 70]. Transfection of HT22 cells with D2^WT^ or D2^T61I^ revealed robust secretion of D2^WT^ into the media, whereas D2^T61I^ secretion was reduced by >80% (Fig. 5i and l). This deficit coincided with the accumulation of RIPA-insoluble D2^T61I^ aggregates (Fig. 5j and m) and diminished RIPA-soluble D2^T61I^ in cell lysates (Fig. 5k and n), consistent with the known propensity of D2^T61I^ to aggregate [13, 71]. Notably, soluble CTSB and CTSL levels were significantly reduced in D2^T61I^-expressing cells compared to D2^WT^ (Fig. 5k, o, and p), consistent with proteomic and enzymatic activity data from D2^WT^ and D2^T61I^ brains. CTSD levels remained unaffected (Fig. 5k and q). We also replicated these findings in transiently transfected HEK293T cells (Fig. S6a to i).

### Lysosomal accumulation of CHCHD2^T61I^ exacerbates lysosomal membrane permeability and vacuolization, impeding αSyn fibril clearance

**Fig. 6:**
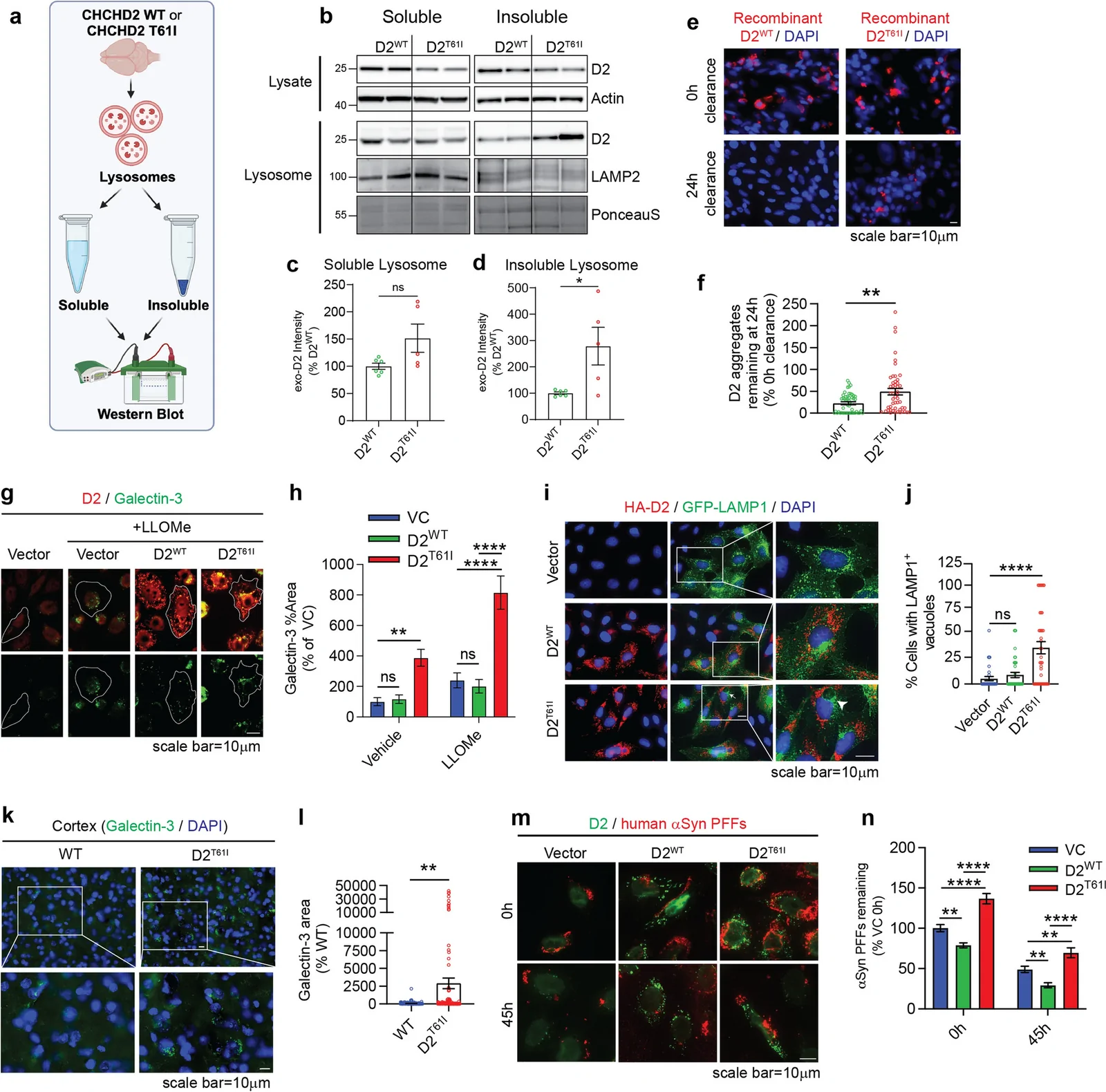
Lysosomal accumulation of CHCHD2^T61I^ promotes lysosomal membrane permeability and vacuolization, impeding αSyn fibril clearance. **a** Schematic of lysosomal isolation and subsequent fractionation into RIPA soluble and insoluble lysates from the cortex of 12-month-old mice. Created with BioRender.com. **b** Representative immunoblots of CHCHD2 and actin from total RIPA soluble and insoluble lysates (upper blots) and CHCHD2 and LAMP2 from lysosomal RIPA soluble and insoluble lysates (lower blots). Quantitative analysis of **c** soluble and **d** insoluble lysosomal CHCHD2 normalized to total lysates (*t* test, *P < 0.05, *ns* not significant, n = 6 D2^WT^, 5 D2^T61I^ mice). **e** Representative images of HT22 cells treated with recombinant CHCHD2^WT^ or CHCHD2^T61I^ and immunostained for CHCHD2 (red) and DAPI (blue). **f** Quantitative analysis of recombinant CHCHD2 aggregates remaining 24 h after treatment (*t* test, **P < 0.005, n = 49 images from 4 wells/condition from 2 experiments). **g** Representative images of HT22 cells transfected with pcDNA3 vector, HA-CHCHD2^WT^, or HA-CHCHD2^T61I^ and treated with 2 μM LLOMe for 2 h and immunostained for CHCHD2 (red) and galectin-3 (green). **h** Quantitative analysis of galectin-3 puncta % area in HT22 cells (two-way ANOVA; post hoc Tukey, **P < 0.01, ****P < 0.0001, *ns* not significant, n = 4 wells/condition from 2 experiments). **i** Representative images of HT22 mouse hippocampal cells co-transfected with pcDNA3 vector, HA-CHCHD2^WT^ or HA-CHCHD2^T61I^ with mGFP-LAMP1 (green) to label lysosomes and immunostained for HA/CHCHD2 (red) and DAPI (blue). White arrows indicate LAMP1+ vacuoles (defined as large vacuolar structures that are positive for mGFP-LAMP1 but negative for DAPI or D2) observed in HA-CHCHD2^T61I^ transfected cells. **j** Quantitative analysis of LAMP1+ vacuoles in transfected HT22 cells (one-way ANOVA; post hoc Tukey, ****P < 0.0001, *ns* not significant, n = 4 wells/condition from 2 experiments). **k** Representative images of cortical tissue from 18-month-old WT and CHCHD2^T61I^ mice immunostained for galectin-3 (green) and DAPI (blue). **l** Quantitative analysis of cortical galectin-3 puncta % area (*t* test, **P < 0.005, *n* = 93–121 images per condition from 4 WT and 5 CHCHD2^T61I^). **m** Representative images of HT22 cells transfected with pcDNA3 vector, HA-CHCHD2^WT^, or HA-CHCHD2^T61I^ and treated with 1 μM of human αSyn PFFs for 4 h, washed, incubated for 0 or 45 h, fixed with 1% triton to solubilize non-fibrillar αSyn, and immunostained for CHCHD2 (green) and human αSyn (red). **n** Quantitative analysis of αSyn intensity (two-way ANOVA; post hoc Tukey, **P < 0.01, ****P < 0.0001, n = 6 wells/condition from 3 experiments). Each dot represents one cell

D2^T61I^ was recently reported to co-localize with the lysosomal marker LAMP1 in dopaminergic neurons derived from human embryonic stem cells [81]. To assess lysosomal accumulation of misfolded D2^T61I^ in the brain, we prepared RIPA-soluble and RIPA-insoluble fractions from isolated lysosomes and total cortical lysates from 10–12-month-old D2^WT^ and D2^T61I^ mouse brains (Fig. 6a). Insoluble D2^T61I^ levels were significantly elevated in lysosomal fractions compared to D2^WT^ (Fig. 6b and d, lower blots), despite reduced D2^T61I^ levels in total lysates (Fig. 6b, upper blots) and comparable soluble D2^WT^ levels in lysosomal fractions (Fig. 6b and c). Lysosomal purity was also verified by comparing different subcellular fractions with known lysosomal and cytosolic markers (Fig. S6j). These results indicate pathological lysosomal accumulation of misfolded D2^T61I^ in the brain, consistent with our proteomics data, implicating impaired lysosomal function (Fig. 3, a to c).

Next, we purified recombinant human D2^WT^ and D2^T61I^ proteins from *E. coli* to evaluate their solubility and aggregation dynamics in the endo-lysosomal pathway. At 4 °C, both proteins existed as monomers and multimers. However, D2^T61I^ monomers were unstable at 37 °C (16 h), forming high molecular weight aggregates that failed to migrate into the SDS stacking gel (Fig. S7a, red asterisk). D2^WT^ monomers, on the other hand, remained relatively stable at 37 °C, with high molecular weight multimers that entered the gel (Fig. S7a). Exposure of HT22 neuroblastoma cells to pre-aggregated (37 °C, 16 h) D2 proteins for 4 h resulted in detectable cellular uptake of both D2^WT^ and D2^T61I^ multimers (Fig. 6e, and Fig. S7b). After the treatment-containing medium was replaced with fresh medium, intracellular D2^WT^ and D2^T61I^ proteins were monitored immediately and after 24 h of chase. Strikingly, D2^T61I^ aggregates displayed markedly greater resistance to degradation than D2^WT^ (Fig. 6e and f). These results suggest that the intrinsic propensity of D2^T61I^ for misfolding and aggregation could be a driving force of mitochondria-lysosome axis dysfunction, leading to impaired degradation of the array of proteins normally destined for lysosomal clearance.

We then evaluated the impact of D2 variants on lysosomal membrane permeability (LMP), as excessive LMP promotes cytosolic leakage of lysosomal content, triggering protein aggregation, autophagy impairment, mitochondrial dysfunction, and cell death [8, 9]. Using galectin-3 puncta, an established marker of LMP [1, 2, 21, 31], we observed a nearly fourfold increase in puncta area in D2^T61I^-transfected HT22 cells compared to D2^WT^ or vector controls under baseline conditions (Fig. 6g and h). Treatment with the lysosomal damaging agent LLOMe further amplified galectin-3 puncta area in D2^T61I^-transfected cells, compared to vector control or D2^WT^ (Fig. 6g and h).

Lysosomal morphology was assessed by co-transfecting HT22 cells with vector control or HA-D2 variants together with GFP-LAMP1 to visualize LAMP1-positive (LAMP1^+^) lysosomes. D2^T61I^ transfection induced a greater than fourfold increase in LAMP1^+^ lysosomal vacuolization compared to controls (Fig. 6i and j), reminiscent of abnormal vacuolar structures observed after αSyn overexpression [4] and in a rat model of PD [37]. In vivo validation confirmed elevated galectin-3 puncta area in aged D2^T61I^ mouse cortical tissues compared to WT littermates (Fig. 6k and l), indicating pathologically high LMP and lysosomal dysfunction.

Given the central role of lysosomes in αSyn clearance and its intercellular prion-like spread in LBDs [31, 72], we hypothesized that D2^T61I^-induced lysosomal dysfunction impedes αSyn fibril degradation. HT22 mouse hippocampal cells transfected with D2 variants were exposed to sonicated human αSyn pre-formed fibrils (PFFs) for 4 h to allow uptake, followed by PBS washout to remove extracellular αSyn PFFs and fresh medium replacement to allow monitoring of cellular clearance of αSyn PFFs over the following 45 h. D2^T61I^-transfected cells retained significantly more pathological human αSyn at 45 h than D2^WT^ or vector control (Fig. 6m and n). A similar trend was observed immediately after the 4 h αSyn uptake phase (0 h) (Fig. 6m and n), indicating greater intracellular αSyn accumulation at the post-uptake baseline. Overexpression of D2^WT^ had opposing effects, with a significant decrease in remaining exogenous PFFs compared to vector control. Similar results were also observed for total human αSyn (without 1% triton during fixation, Fig. S7c and d), and αSyn uptake into cells was confirmed via Z-stack imaging (Fig. S7e). Cells not treated with human αSyn PFFs showed no immunoreactivity with the human-specific αSyn antibody (Fig. S7f). These results indicate that D2^T61I^ is associated with reduced apparent intracellular clearance relative to D2^WT^, whereas D2^WT^ promotes the apparent clearance of pathological αSyn.

### CHCHD2^WT^ mitigates, while CHCHD2^T61I^ exacerbates, age-related ultrastructural abnormalities in mitochondria and lysosomes

**Fig. 7:**
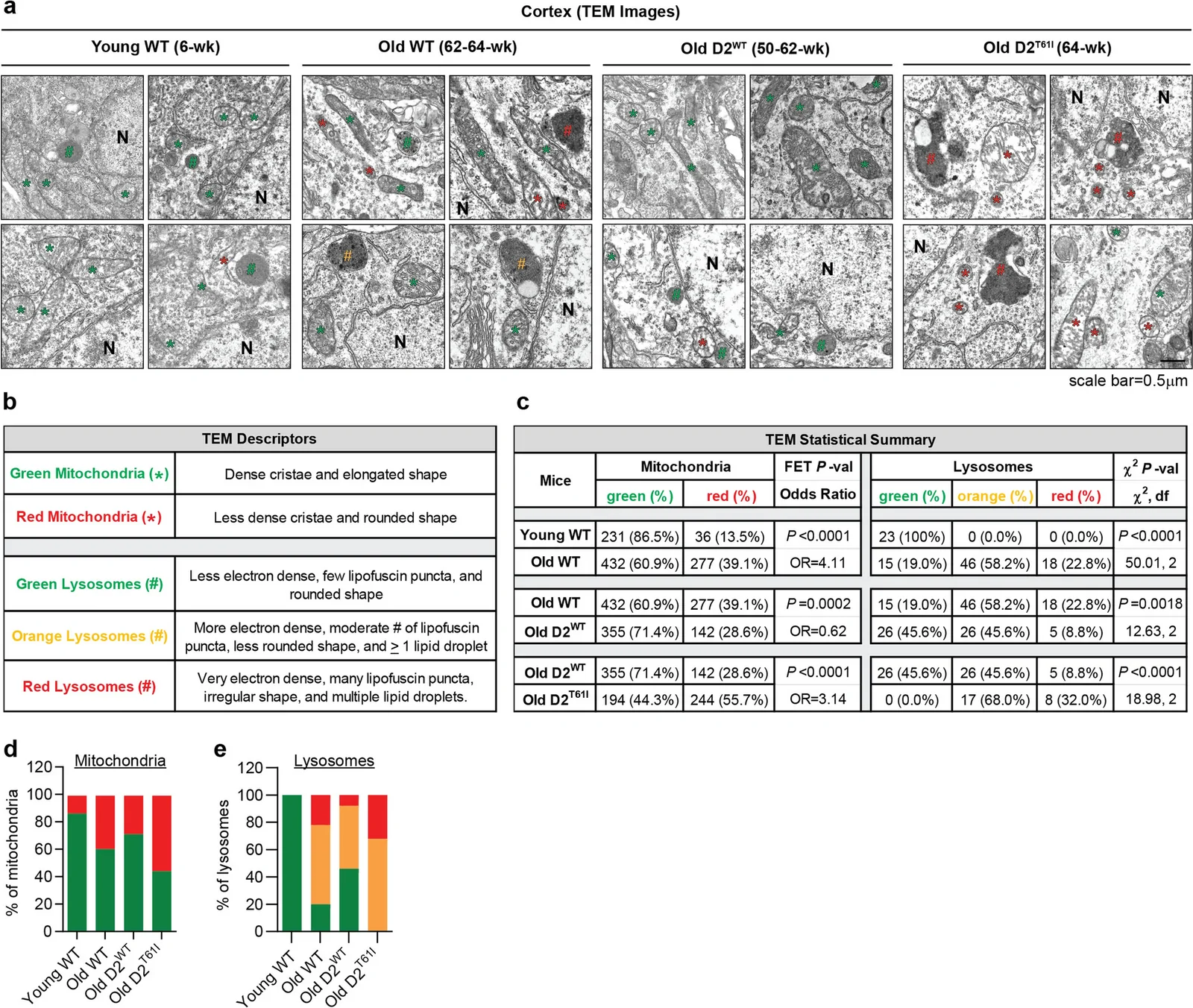
CHCHD2^WT^ decelerates and CHCHD2^T61I^ accelerates age-related ultrastructural abnormalities in mitochondria and lysosomes. **a** Representative TEM images of cortical tissues from 6-week-old WT (young) and 12- to 13-month-old WT, CHCHD2^T61I^, and CHCHD2^WT^ mice (old). *N* nucleus. Mitochondria are labeled with asterisks (*), and lysosomes with hashes (#). **b** Table of TEM descriptors categorizing green and red mitochondria, as well as green, orange, and red lysosome ultrastructure. **c** Contingency tables summarizing statistical results (Fisher’s exact test and *χ*^2^ analysis) of mitochondrial and lysosomal categories from TEM images. **d**, **e** Stacked bar graphs summarizing differences in mitochondrial and lysosomal categories between young WT, old WT, old CHCHD2^WT^ and old CHCHD2^T61I^ mice (n = 8 cells from 2 young WT, 26 cells from 6 old WT, 18 cells from 4 old CHCHD2^WT^, and 11 cells from 3 old CHCHD2^T61I^)

To evaluate age- and D2-dependent structural changes in mitochondria and lysosomes in vivo, we performed transmission electron microscopy (TEM) on cortical tissues from young WT (6-week-old) and old mice (12–13-month-old: WT, D2^WT^, and D2^T61I^) and sorted mitochondria and lysosomes in neuronal cell bodies into multiple classifications (Fig. 7a and Fig. S7g). Mitochondrial and lysosomal ultrastructure classifications are outlined in Fig. 7b and a statistical summary of TEM analysis is presented in Fig. 7c.

With regard to mitochondrial ultrastructure, aged WT mitochondria exhibited pronounced structural deterioration, including reduced cristae density and rounded morphology (labeled as red asterisks), compared to young WT mitochondria, which predominantly displayed healthy elongated shapes with dense cristae (> 86%, labeled as green asterisks) (Fig. 7a to d). Strikingly, aged D2^WT^ mitochondria showed a significantly lower proportion of abnormal mitochondria compared to age-matched WT (Fig. 7a to d), indicating a protective role for D2^WT^ against age-related mitochondrial instability. By contrast, aged D2^T61I^ mitochondria displayed severe ultrastructural abnormalities, with far greater frequency of unhealthy mitochondria than both aged D2^WT^ and WT groups (Fig. 7a to d), consistent with prior reports of mitochondrial ultrastructural abnormalities in D2^T61I^ knock-in mice [19].

With regard to lysosomal ultrastructure, we classified lysosomal integrity as green, orange, and red, representing a healthy to less healthy appearance, according to factors of electron density, lipofuscin puncta, lipid droplets, and lysosomal shape (Fig. 7b). While 100% of young WT lysosomes were classified as ‘green’ (rounded shaped, few lipofuscin puncta, and less electron dense, labeled with hashes), aged WT lysosomes predominantly (~ 80%) exhibited orange or red classification (Fig. 7a to c, and e), reflecting age-related lysosomal dysfunction linked to the accumulation of lipofuscin granules and impaired waste clearance, known to promote cellular dysfunction [69]. Aged D2^WT^ lysosomes retained significantly higher proportions of healthy (green) structures than aged WT lysosomes (Fig. 7a to c, and e), indicating that D2^WT^ protects against the age-related loss of lysosomal integrity. Conversely, no D2^T61I^ lysosomes were classified as healthy (green), with this group showing exacerbated structural abnormalities compared to both aged D2^WT^ and WT (Fig. 7a to c, and e). Notably, aged D2^T61I^ lysosomes displayed electron-dense, swollen structures with irregular shapes and frequent lipid droplet accumulation (Fig. 7a), consistent with severe lysosomal dysfunction.

Together, these ultrastructural changes in the mitochondrial-lysosomal axis demonstrate that D2^T61I^ exacerbates age-related mitochondrial fragmentation and lysosomal abnormalities, while D2^WT^ protects the axis from this fate.

## Discussion

The D2 mitochondrial protein plays a critical role in LBDs, particularly PD [23, 32, 55, 67, 78]. While the D2^T61I^ mutation is linked to mitochondrial dysfunction and cytoplasmic phospho-αSyn-containing insoluble aggregates [27, 33, 71], the broader mechanistic impact of this mutation on the mitochondria-lysosome axis has remained unclear. Furthermore, the potential protective role of D2^WT^ against age-related neurodegenerative changes, as well as its relationship to lysosomal dysfunction, a hallmark of PD pathogenesis [21, 31, 63, 77], has not been explored, particularly regarding cathepsin-mediated protein degradation.

In this study, we advance our understanding of D2’s role in brain aging, proteinopathy, and neurodegeneration through several key discoveries. We demonstrate an age-dependent decline in D2 expression in the human brain, potentially increasing susceptibility to LBDs, consistent with mitochondrial dysfunction and dopaminergic neuron loss in aged D2-deficient mice [66]. Strikingly, D2^WT^ mice exhibited robust neuroprotection against age-related degeneration compared to WT littermates and D2^T61I^ mice, including preserved mitochondrial and lysosomal ultrastructure, reduced neuroinflammation, enhanced dopaminergic neuron survival, and diminished insoluble protein accumulation. These findings suggest that age-related D2 decline may heighten vulnerability to neurodegeneration, while maintaining or enhancing D2 function could counteract these effects. By contrast, the D2^T61I^ mutation accelerates brain aging via the mitochondria-lysosome axis, exacerbating axonal degeneration and cognitive deficits. These results align with and expand upon prior observations of motor dysfunction, dopaminergic neuron loss, and pathological αSyn accumulation in D2^T61I^ mice [19, 33, 71].

Proteomic profiling revealed divergent effects of D2^WT^ and D2^T61I^ on protein homeostasis: D2^WT^ reduced the ratio of insoluble to soluble proteins, whereas D2^T61I^ increased it, suggesting that D2^WT^ enhances proteostatic clearance while D2^T61I^ promotes protein aggregation. Pathway analysis of the proteomes implicated the lysosomal CLEAR pathway, with D2^T61I^ brains showing marked downregulation of lysosomal proteins and CLEAR signaling. This was further supported by interactome and PLA data, which revealed the preferential binding of D2^T61I^ over D2^WT^ to CTSB and CTSL. Correspondingly, D2^T61I^ expression reduced CTSB and CTSL levels in vitro and activities in vivo, providing a novel mechanism by which this mutation impairs the lysosomal clearance of substrates like αSyn. Notably, a familial PD patient with the D2^T61I^ mutation exhibited comorbid αSyn, amyloid, and tau pathologies [27], mirroring the various forms of proteinopathy linked to lysosomal failure. The correlation between reduced D2 and CTSB levels in LBD patient cortices further underscores the functional relationship between D2 and lysosomal proteases, establishing a direct link to the mitochondrial-lysosomal axis.

The selective interaction of D2^T61I^ with CTSB and CTSL, but not CTSD, holds particular significance given CTSB’s genetic association with PD risk [7, 30, 53] and the major roles of lysosomal CTSB and CTSL in αSyn clearance [30, 48, 49, 65]. A recent study showed that genetically predicted increases in CTSB levels correlate with a significantly reduced risk for PD [44], in line with our observation in LBD patient brains. Experimentally, CTSB and CTSL cleave αSyn in its amyloid region, which prevents fibril formation, whereas the aspartyl protease CTSD incompletely degrades αSyn, still leading to fibril formation [49]. CTSB and CTSL, but not CTSD, also degrade αSyn preformed fibrils (PFFs) [49]. In iPSC-derived dopaminergic neurons, CTSB not only promotes fibrillar αSyn clearance but also augments lysosomal function and GBA activity [30], while CTSL overexpression reduces αSyn aggregation [48]. Reciprocally, αSyn mutations (A53T and E46K) impair CTSL-mediated cleavage [48, 65], suggesting that lysosomal protease dysfunction contributes to mutant αSyn pathogenesis. Furthermore, overexpression of αSyn impairs the trafficking of cathepsins, including CTSB, and iPSC-derived dopaminergic neurons from PD patients with *SNCA* triplication or the αSyn^A53T^ mutation exhibit decreased total and lysosomal levels of CTSB, CTSD, and CTSL [18], consistent with our finding of reduced CTSB and CTSL in LBD cortex and by D2^T61I^ expression. Hence, the inhibitory action of D2^T61I^ on CTSB and CTSL may help explain the accumulation of αSyn observed in human PD patients and mouse models of D2^T61I^ [19, 33, 71]. While the underlying basis for the specificity for CTSB and CTSL, as opposed to CTSD, is unclear, we propose that lysosomal accumulation of misfolded D2^T61I^ disrupts CTSB and CTSL trafficking and stability, akin to the effects of misfolded αSyn [18]. On the other hand, reduction in endogenous D2 level appears more highly correlated with CTSB than CTSL levels.

The central role of D2 in the mitochondria-lysosome axis is clear in our TEM studies. D2^WT^ protects against age-related mitochondrial and lysosomal structural abnormalities, whereas D2^T61I^ accelerates mitochondrial cristae loss, lysosomal vacuolization, and lipofuscin accumulation. These mitochondrial effects align with the known role of D2 in maintaining mitochondrial cristae structure and function [32, 42, 51], as well as the perturbations in mitochondrial fatty acid β-oxidation / activation, branched amino acid degradation, and the L-carnitine shuttle, as seen in this study. In contrast, the ultrastructural effects of D2 on lysosomes are completely novel. While age-related deterioration of lysosomal function is well-documented, with the accumulation of lipofuscin granules being a hallmark of aging lysosomes [69], our findings indicate that D2 plays a crucial role in maintaining lysosomal health in the aging brain, with its dysfunction contributing to lysosomal aging and defects observed in LBDs, including lysosomal lipofuscin granule formation, lipid droplet formation, vacuolization, and irregular enlarged morphology [16, 21, 31, 63, 77]. We hypothesize that D2 dysfunction originates in mitochondria, propagating to lysosomes where misfolded D2^T61I^ accumulates, impairing secretory clearance. However, D2’s potential role in a causal relationship between mitochondrial and lysosomal abnormalities still remains to be determined.

Overall, this study establishes D2 as a critical regulator of the mitochondria-lysosome axis, with dysfunction driving brain aging and LBD pathogenesis. The contrasting phenotypes of D2^WT^ and D2^T61I^ mice provide mechanistic insights into disease pathways and therapeutic opportunities. Given the convergence of mitochondrial and lysosomal dysfunction in LBDs, therapeutic strategies targeting both organelles simultaneously, such as stabilizing D2 expression, preventing its misfolding, or disrupting interactions between misfolded D2 and CTSB / CTSL, may offer synergistic benefits. These approaches could simultaneously preserve mitochondria and lysosomes during aging and LBD pathogenesis by addressing a root cause of proteostatic failure.

## Supplementary Information

Below is the link to the electronic supplementary material.

Supplementary file1 (PDF 107383 kb)

## Data Availability

All data needed to evaluate the conclusions of the paper are present in the paper and/or the Supplementary Materials.
