# Supplementary material for: CHCHD2 mitigates age-associated proteostatic collapse by maintaining homeostasis of the mitochondria-lysosome axis
Source: Acta Neuropathol. 2026 Oct 13;152(1):54. doi: 10.1007/s00401-026-03093-4 (PMC13661657; doi:10.1007/s00401-026-03093-4)
Supplement: Supplementary file 1 [file 401_2026_3093_MOESM1_ESM.pdf]

Supplemental Figure S1

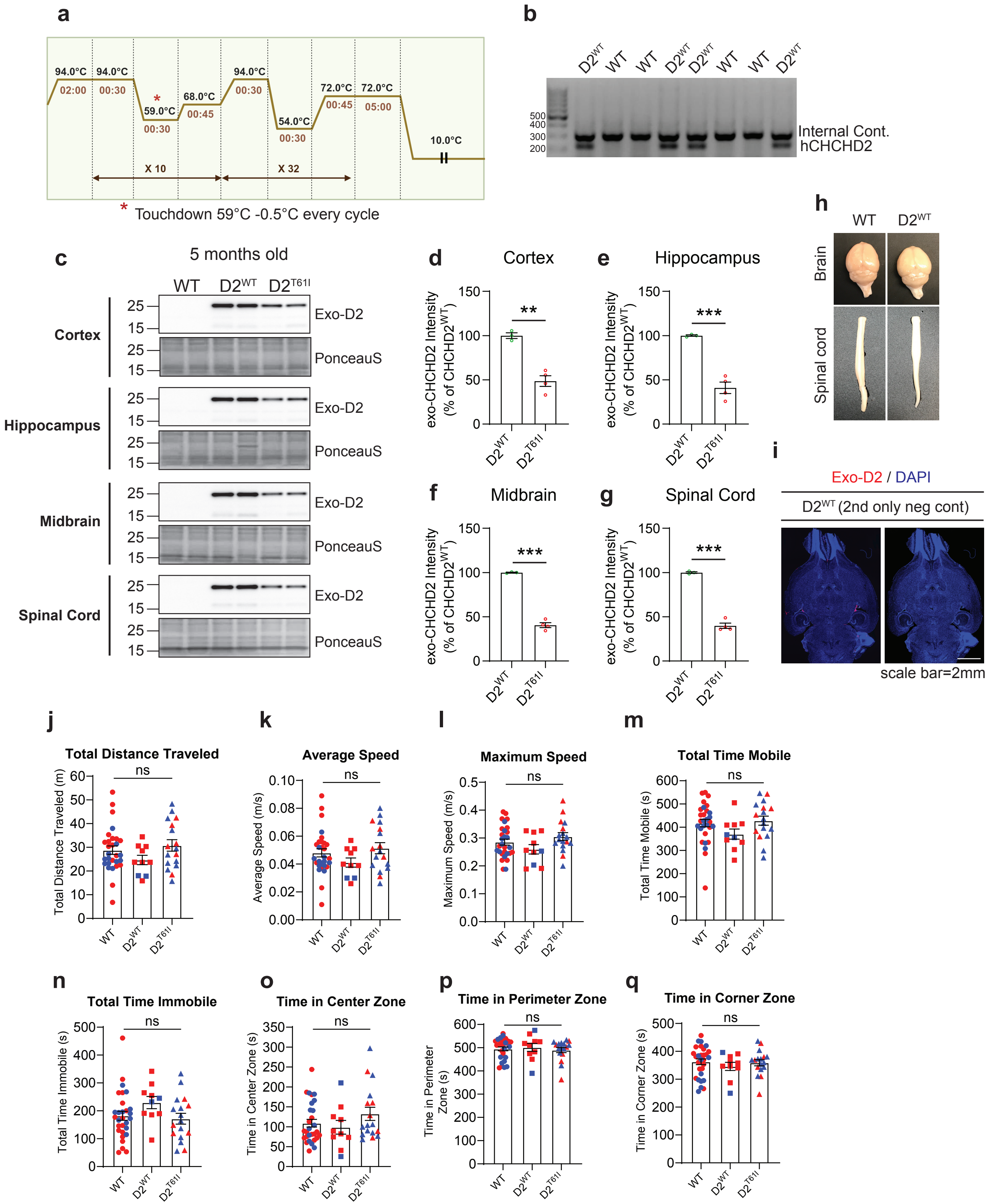

Supplemental Figure S2

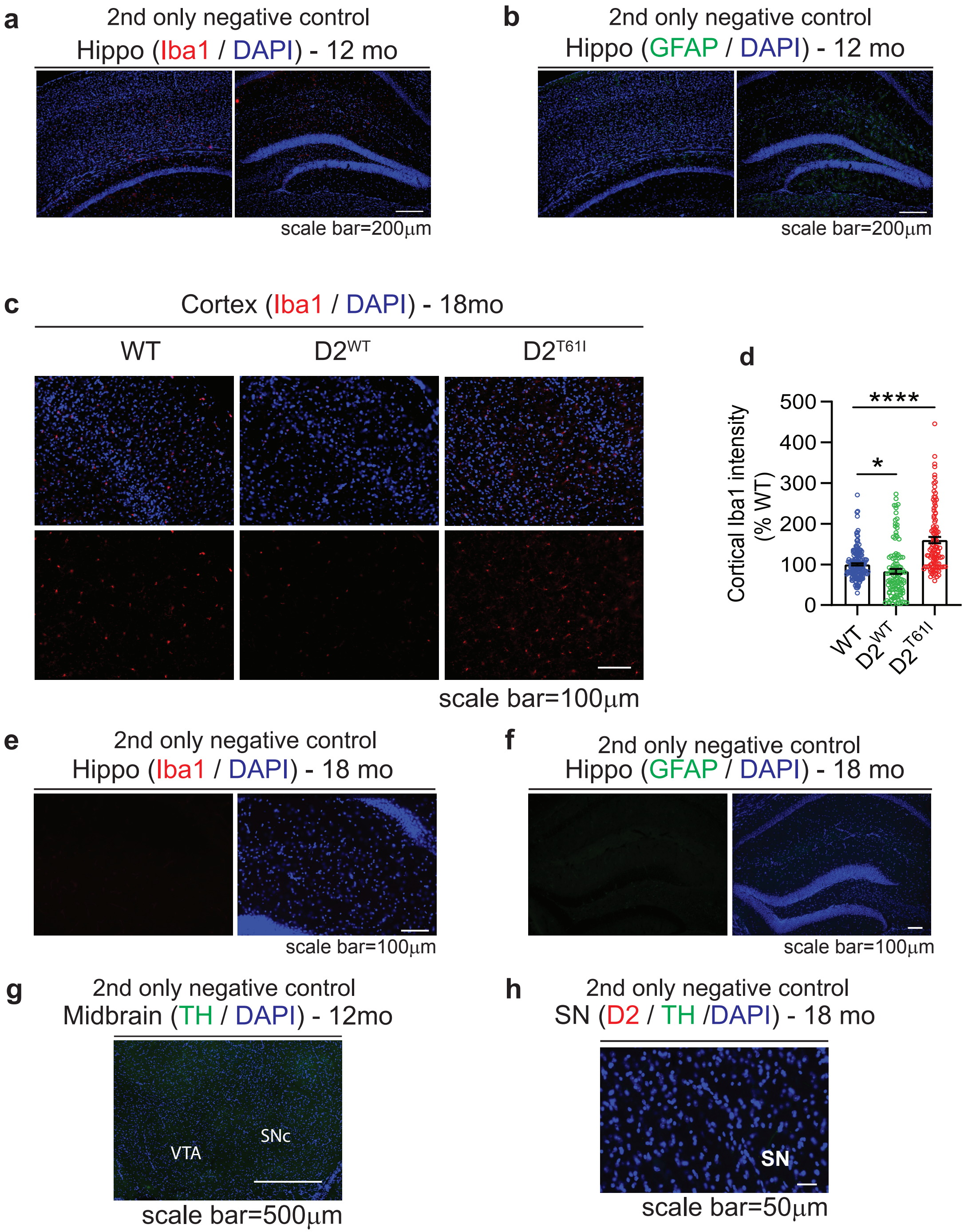

Supplementary Figure S3

a

Significantly altered insoluble vs soluble proteins ($D2^{T61I}$ vs WT)				
**Mouse Genotype**	**Insoluble proteins (increased)**	**Soluble proteins (increased)**	**Odds Ratio (Insoluble vs soluble)**	**FET *P*-value**
$CHCHD2^{T61I}$	245	43	OR=23.4 ($D2^{T61I}$ vs WT)	<0.0001
WT	28	115		

b

**Lysosomal proteins reduced in $D2^{T61I}$ vs $D2^{WT}$ brains**		
**Protein**	***P*-value**	**Major function**
LAMTOR3	8.19E-06	Lysosome membrane mTorc1 docking
Rab39A	0.000486	Lysosome/phagosome maturation
ARL8B	0.018127	Cargo trafficking to lysosomes
LAMP-2	0.024648	CMA and autophagy
HEXA	0.033102	Lysosomal hydrolase
AP3-S2	0.038572	Membrane transport to lysosomes
Cathepsin B	0.040254	Major lysosomal protease
Cathepsin L	0.044719	Major lysosomal protease
AP3-S1	0.048935	Membrane transport to lysosomes

c

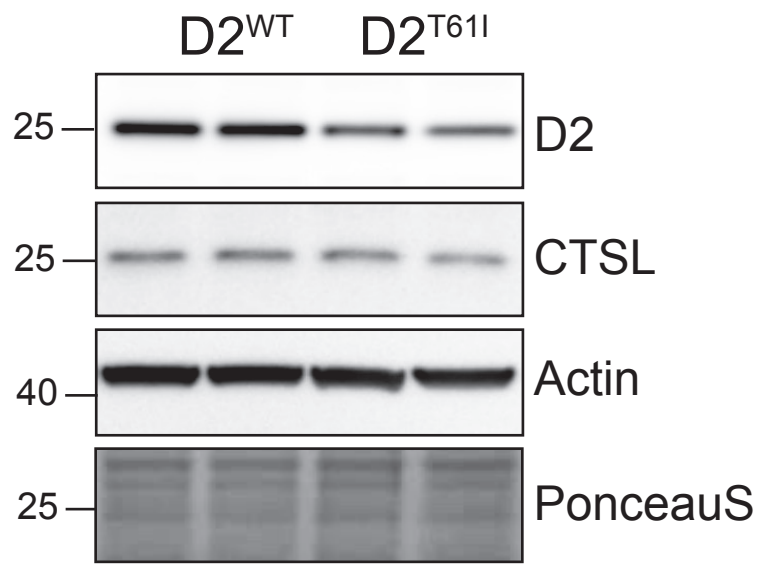

d

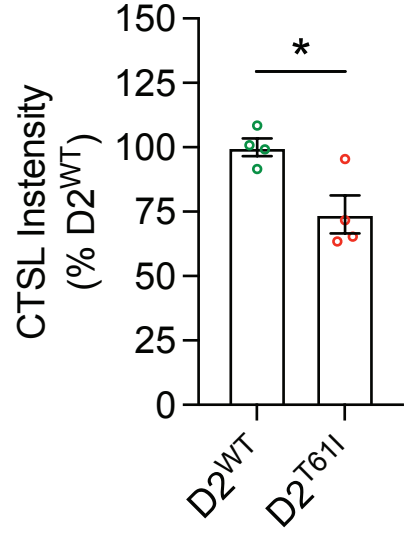

Supplementary Figure S4

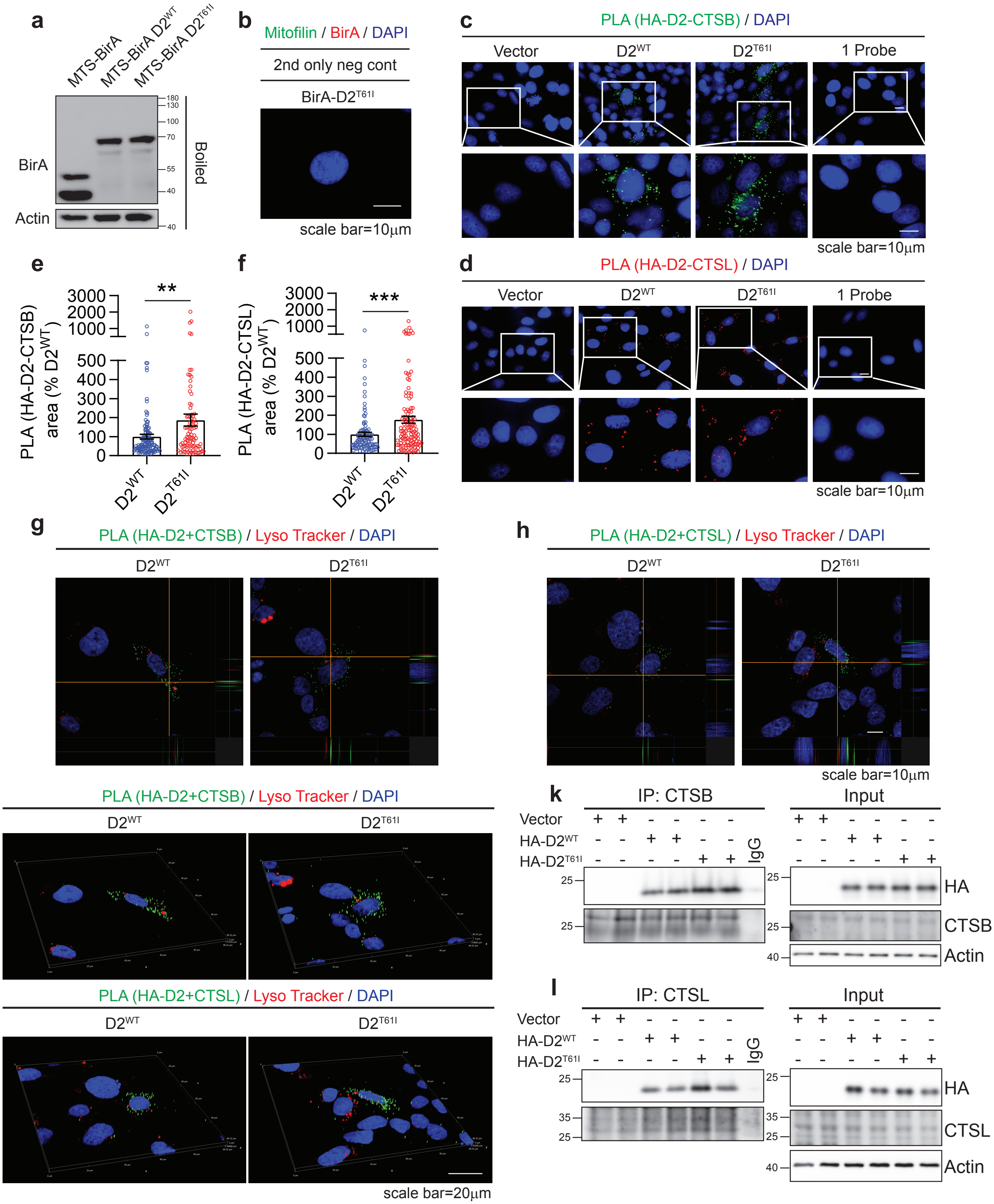

Supplementary Figure S5

	Case Number	Primary Neuropathologic Diagnosis	Secondary Neuropathologic Diagnosis	Tertiary Neuropathologic Diagnosis	PMI (hr)	Age at Onset	Age at Death/Bx	Duration (years)	ApoE	Race/Sex
p1	A86-46	Control			na		65		E3/3	wm
p3	OS99-08	Control			3		74		E3/3	wf
p8	E10-142	Control	Microinfarct-Hp	NFT-Braak stage II	5.5		94		E3/3	wm
p10	E16-45	Control	Braak I		2.5		70		E3/3	bm
p2	OS00-23	Control			11		68		E3/3	bf
p3	OS02-35	Control			6		75		E3/3	wf
p8	E06-41	Control			10		57		E3/3	wm
p9	E08-101	Control	Large cerebral hemorrhage		11.5		78		E3/3	wf
p15	OS03-395	LBD-neocortical	Status post-pallidotomy		10	46	78	32	E3/3	wm
p16	OS03-396	LBD-neocortical			13	52	64	12	E2/3	wm
p17	OS00-18	LBD-neocortical	Status Post Gamma-Knife Radiosurgery		3	62	69	7	E3/3	wm
p23	E16-138	LBD-neocortical	AD-possible	Braak II	10.5	48	69	21	E3/3	wm
p28	OS99-05	LBD-neocortical	TDP-43 incl-Hp,Am		19	53	75	22	E2/3	wm
p27	OS97-11	LBD-neocortical	AD-probable	TDP-43 incl-Hp,Ent; Microinfarcts-BG,Cb	8	79	85	6	E3/4	bm
p34	E09-136	LBD-neocortical			22	61	72	11	E3/4	wf
p35	E12-14	LBD-neocortical	AD-possible	TDP-43 incl-Amyg,Ento	15	52	60	8	E4/4	wm
p36	E12-97	LBD-limbic	TDP-43 incl-Hp,Ent		13	92	97	5	E3/3	wf

Supplementary Figure S6

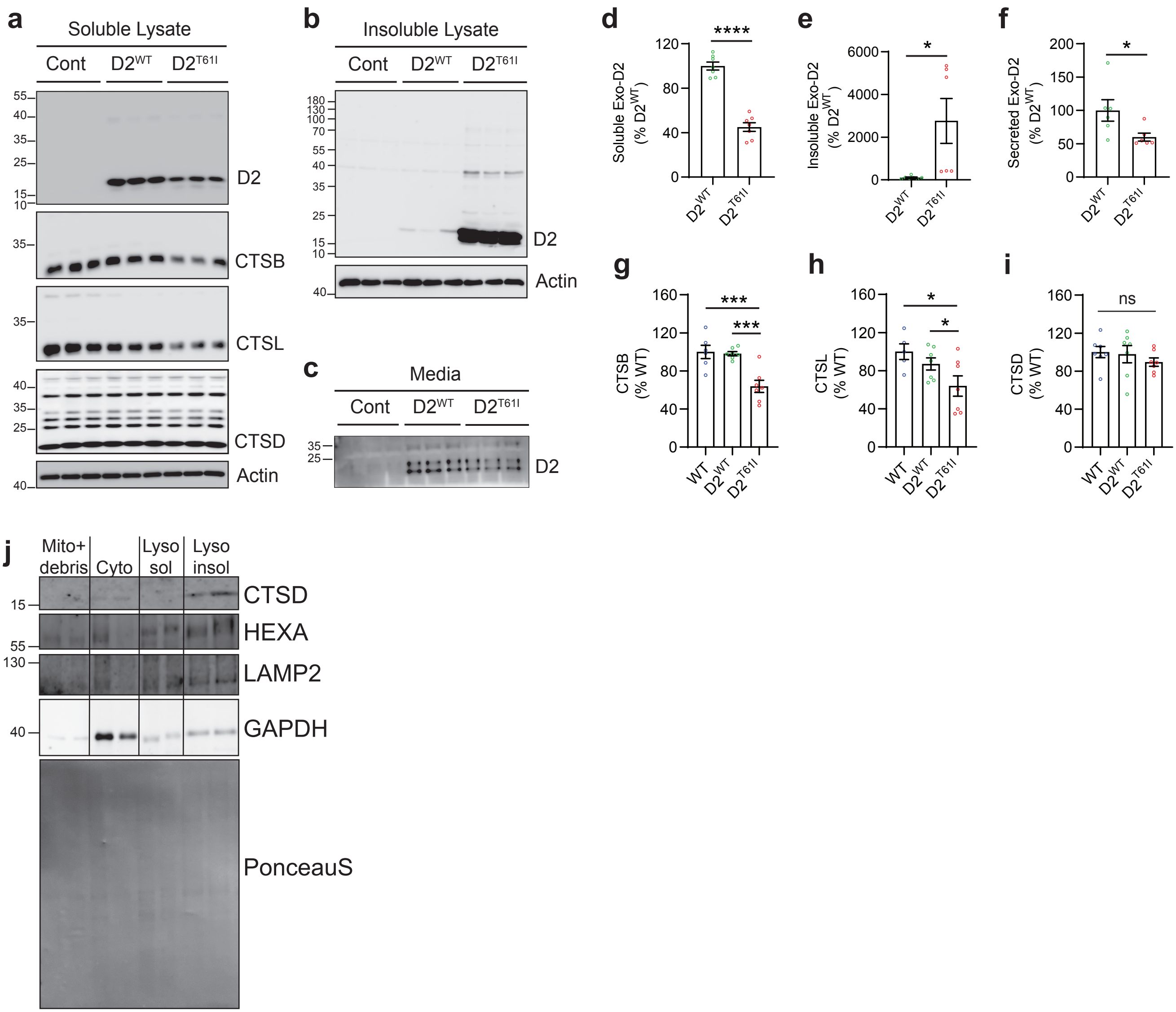

Supplementary Figure S7

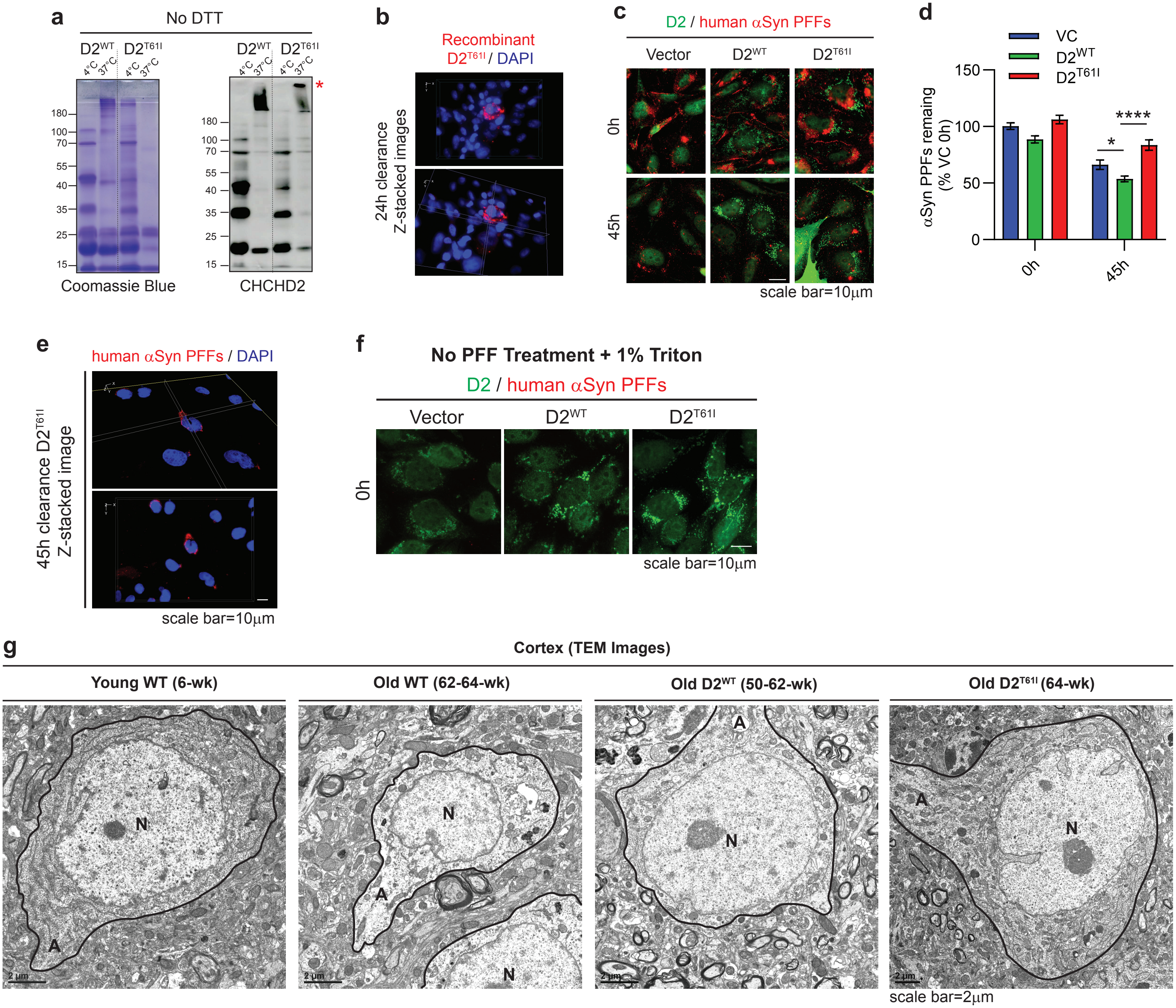

Supplementary Figure Legends

Fig. S1 Characterization of CHCHD2^{WT} mice

(**a**) PCR protocol for CHCHD2 genotyping (forward primer: 5' CAG CAG CCT TGC CTC TAT GA 3', reverse primer: 5' GTA CAA GAA AGT TGG GTA GGC CAA T 3'). (**b**) Representative DNA agarose gel of CHCHD2 genotyping with ~220bp human CHCHD2 band and ~300bp internal positive control. (**c**) Representative immunoblots of RIPA-soluble brain lysates (RPI RIPA Buffer, R26200) blotted for CHCHD2. Quantitative analysis of exogenous CHCHD2 levels in the cortex (**d**), hippocampus (**e**), midbrain (**f**), and spinal cord (**g**) of 5-month-old mouse brains (t-test, ***P*<0.01, ****P*<0.001, n=4 mice/genotype). (**h**) Representative images of whole brains and spinal cords from 12-month-old WT and CHCHD2^{WT} mice. (**i**) Secondary only negative control images for Fig. 1i. (**j-q**) Quantitative analysis of open field testing in 18-month-old WT, CHCHD2^{WT}, and CHCHD2^{T61I} mice (one-way ANOVA; post hoc Tukey, ns=not significant, n=29 WT, 10 CHCHD2^{WT}, and 17 CHCHD2^{T61I}). Females are in red, and males in blue.

Fig. S2 Age-related changes in CHCHD2^{WT} vs CHCHD2^{T61I} mice

(**a**) Secondary only negative control image for Fig. 2a. (**b**) Secondary only negative control image for Fig. 2c. (**c**) Representative images of cortical tissues from 18-month-old WT, CHCHD2^{WT}, and CHCHD2^{T61I} mice immunostained for Iba1 (red) and DAPI (blue). (**d**) Quantitative analysis of cortical Iba1 intensity (one-way ANOVA; post hoc Tukey, **P*<0.05, *****P*<0.0001, n=105-183 images per condition from 8 WT, 4 CHCHD2^{WT}, and 5 CHCHD2^{T61I}). (**e**) Secondary only negative control images for Fig. 2e. (**f**) Secondary only negative control images for Fig. 2g. (**g**) Secondary only negative control images for Fig. 2i. (**h**) Secondary only negative control image for Fig. 2m.

Fig. S3 Reduction of lysosomal proteins in CHCHD2^{T61I} vs CHCHD2^{WT} vs mice

(**a**) Contingency table listing the number of significantly altered RIPA insoluble and soluble proteins in 10-month-old CHCHD2^{T61I} cortex compared to WT littermates [31]. FET=Fisher's exact test. (**b**) Table summarizing lysosomal proteins that are significantly reduced in CHCHD2^{T61I} compared to CHCHD2^{WT} mouse cortex (Welch's t-test *P*-values are indicated). Major functions of the proteins are indicated. (**c**) Representative immunoblots for CHCHD2, CTSL, and actin of RIPA-soluble cortical lysates from 10 to 12-month-old mice. (**d**) Quantitative analysis of RIPA-soluble cortical CTSL levels normalized to actin (t-test, **P*<0.05, n=4 mice/genotype).

Fig. S4 Characterization of MTS-BirA variants

(**a**) Representative immunoblots for BirA and actin in HT22 cells transiently transfected with MTS-BirA, MTS-BirA-CHCHD2^{WT}, or MTS-BirA-CHCHD2^{T61I}. (**b**) Secondary only negative control for Fig. 4b. (**c,d**) Representative images of PLA (HA-CHCHD2 / CTSB interaction, green) and (**F**) PLA (HA-CHCHD2 / CTSL interaction, red) in HT22 cells transfected with pcDNA3 vector, HA-CHCHD2^{WT}, or HA-CHCHD2^{T61I} without LysoTracker. (**e**) Quantitative analysis of PLA (HA-CHCHD2 / CTSB) puncta % area (t-test, ***P*<0.005, n=4 wells/condition from 2 experiments) and (**f**) PLA (HA-CHCHD2 / CTSL) PLA puncta % area (t-test, ****P*<0.001, n=4 wells/condition from 2 experiments). (**g-j**) Representative Z-stack images from Fig. 4e,f indicating a lack of PLA colocalization with LysoTracker. (**k,l**) Representative co-immunoprecipitation blots for either CTSB or CTSL pulldown and immunoblotting for HA-CHCHD2 from 2 experiments each.

Fig. S5 Control and LBD case information

Table includes pathological diagnosis, postmortem interval (PMI), age at disease onset, age at death, duration of disease, APOE genotypes, race (w=white, b=black), and sex (m=male, f=female).

Fig. S6 CHCHD2^{T61I} but not CHCHD2^{WT} reduces CHCHD2 secretion, increases insoluble CHCHD2, and lowers Cathepsin B and L levels

(a and **d)** Representative immunoblots and quantitative analysis of RIPA soluble CHCHD2, (**b** and **e**) RIPA insoluble CHCHD2, and (**c** and **f**) secreted CHCHD2 from HEK293T cells transfected with pCDNA3, HA-CHCHD2^{WT}, or HA-CHCHD2^{T61I} (t-test, **P*<0.05, *****P*<0.0001, n=7 wells/condition from 2 experiments). (**g-i**) Quantitative analysis of cathepsin levels in HEK293T cells (one-way ANOVA; post hoc Tukey, **P*<0.05, ****P*<0.001, ns=not significant, n=7 wells/condition from 2 experiments). (**j**) Representative immunoblots for lysosomal markers (CTSD, HEXA, and LAMP2) and GAPDH cytosolic marker from different fractions of WT mouse cortex to determine the purity of lysosomal isolation.

Fig. S7 Aggregation and cellular uptake of CHCHD2^{WT} and CHCHD2^{T61I}

(**a**) Representative Coomassie Blue stained gels (left) and CHCHD2 immunoblots (right) for CHCHD2^{WT} and CHCHD2^{T61I} recombinant proteins incubated at 4°C or 37°C for 16 hours with shaking, the latter to induce protein aggregation. The red asterisk indicates CHCHD2^{T61I} aggregates that fail to enter the stacking gel. (**b**) Representative Z-stacked images of HT22 cells treated with aggregated recombinant CHCHD2^{T61I} protein and subjected to cellular clearance for 24 hours, then stained for CHCHD2 (red) and DAPI (blue) to visualize cellar CHCHD2^{T61I} internalization and remaining aggregates. (**c**) Representative images of HT22 cells transfected with pcDNA3 vector, HA-CHCHD2^{WT}, or HA-CHCHD2^{T61I} and treated with 1μM of human αSyn PFFs for 4 hours, washed, incubated for 0 or 45 hours, and immunostained for CHCHD2 (green) and human αSyn (red). (**d**) Quantitative analysis of αSyn intensity (two-way ANOVA; post hoc Tukey, **P*<0.05, *****P*<0.0001, n=4 wells/condition from 2 experiments). (**e**) Representative Z-stack images for human αSyn (red) and DAPI (blue) indicating uptake of exogenous αSyn PFFs. (**f**) Representative images of HT22 cells not treated with αSyn PFFs from Fig. 6m. (**g**) Representative TEM images of whole cortical neuronal cell bodies. N=nucleus, A=axon.

Table S1: Proteins significantly altered in D2-T61I vs D2-WT cortex

Protein names	Gene names	Ratio (D2-T61I / D2-WT)	P-value
UPF0585 protein C16orf13 homolog	0610011F06Rik	1.326299108	0.010359394
ATP-binding cassette sub-family B member 7, mitochondrial	Abcb7	1.203175786	0.022583838
Alpha/beta hydrolase domain-containing protein 17B	Abhd17b	1.50975E-08	0.000255783
Actin-binding LIM protein 1	Ablim1	2.517936229	0.048450303
Isobutyryl-CoA dehydrogenase, mitochondrial	Acad8	1.448341659	0.044229429
Short-chain specific acyl-CoA dehydrogenase, mitochondrial	Acads	1.476997819	0.004134385
Short/branched chain specific acyl-CoA dehydrogenase, mitochondria	Acadsb	1.254387914	0.032871538
Acyl-coenzyme A thioesterase 13;Acyl-coenzyme A thioesterase 13, N-terminally processed	Acot13	1.381254697	0.031782965
Long-chain-fatty-acid--CoA ligase ACSBG1	Acsbg1	1.203137699	0.042522908
Long-chain-fatty-acid--CoA ligase 1	Acsl1	1.176544009	0.018760596
Long-chain-fatty-acid--CoA ligase 3	Acsl3	1.287666394	0.042478291
Actin, cytoplasmic 2;Actin, cytoplasmic 2, N-terminally processed	Actg1	0.236143783	0.045180003
Actinin alpha 4	Actn4	5.240916083	0.009013919
Acylphosphatase-2	Acyp2	2.090519627	0.016093144
Uncharacterized aarF domain-containing protein kinase 1	Adck1	0.226709377	0.028226477
Putative adenosylhomocysteinase 2	Ahcyl1	1.171128297	0.041540869
Alcohol dehydrogenase [NADP(+)]	Akr1a1	1.198274982	0.04443207
AMP deaminase 2	Ampd2	1.471674405	0.010659926
Annexin A11;Annexin	Anxa11	0.757002238	0.023360668
AP-3 complex subunit beta-2	Ap3b2	1.174777168	0.017464125
AP-3 complex subunit mu-1	Ap3m1	3.323964463	0.040962733
AP-3 complex subunit sigma-1	Ap3s1	0.428225778	0.048935477
AP-3 complex subunit sigma-2	Ap3s2	0.296837721	0.038572064
Amyloid beta A4 precursor protein-binding family B member 1	Apbb1	5.092561735	0.008420681
APP-like protein 2	Aplp2	1.358024327	0.042144194
Apolipoprotein E	Apoe	1.537595737	0.022465159
Arfaptin-2	Arfip2	0.645034403	0.018127029
ADP-ribosylation factor-like protein 1	Arl1	3.784749231	0.040708098
ADP-ribosylation factor-like protein 6	Arl6	5.340677428	0.006323627
ADP-ribosylation factor-like protein 8B	Arl8b	0.566690185	0.018141751
Armadillo repeat-containing protein 1	Armc1	1.2290625	0.041082092

**Protein names**	**Gene names**	**Ratio (D2-T61I / D2-WT)**	**P-value**
Argininosuccinate synthase	Ass1	1.45915234	0.010719227
Autophagy-related protein 2 homolog B	Atg2b	2.35755E-08	0.000150237
Sarcoplasmic/endoplasmic reticulum calcium ATPase 2	Atp2a2	0.825145965	0.039640959
BAI1-associated protein 3	Baiap3	62881666.67	0.035339755
3(2),5-bisphosphate nucleotidase 1	Bpnt1	81126000	0.001062398
BRI3-binding protein	Bri3bp	1.364990246	0.040846328
Voltage-dependent L-type calcium channel subunit beta-3	Cacnb3	0.792792793	0.038494311
Calretinin	Calb2	1.737302076	0.028171816
Calumenin	Calu	66268500	0.000165961
Adenylyl cyclase-associated protein 2;Adenylyl cyclase-associated pro	Cap2	0.853593747	0.040942395
Caskin-1	Caskin1	1.23177887	0.017403107
Carbonyl reductase [NADPH] 3	Cbr3	0.88497419	0.018770112
Coiled-coil domain-containing protein 136	Ccdc136	64018666.67	0.000519087
OX-2 membrane glycoprotein	Cd200	1.574058874	0.038121818
CDC42 small effector protein 2	Cdc42se2	8.88491E-09	0.000940064
Ceramide synthase 1	Cers1	1.55037E-08	0.002593297
Mitochondrial intermembrane space import and assembly protein 40	Chchd4	5.366355767	0.008034139
Neural cell adhesion molecule L1-like protein;Processed neural cell adhesion molecule L1-like protein	Chl1	1.415821334	0.0467087
Anamorsin	Ciapin1	1.261545456	0.043562004
C-type lectin domain family 2 member L	Clec2l	3.898502592	0.030278397
Calmin	Clmn	0.194061024	0.015810276
Calsyntenin-1;Soluble Alc-alpha;CTF1-alpha	Clstn1	1.51423E-08	0.000601108
Clavesin-2	Clvs2	0.333825561	0.020492329
Citrate lyase subunit beta-like protein, mitochondrial	Clybl	1.549534199	0.015699921
Carboxymethylenebutenolidase homolog	Cmbl	1.508169843	0.031625116
Ciliary neurotrophic factor receptor subunit alpha	Cntfr	0.71685059	0.004934155
Cytochrome c oxidase assembly factor 3 homolog, mitochondrial	Coa3	4.355168829	0.047991821
COMM domain-containing protein 9	Commd9	0.215091986	0.012255102
Ubiquinone biosynthesis O-methyltransferase, mitochondrial	Coq3	83165000	0.004888398
Coactosin-like protein	Cotl1	1.292249296	0.02080248
Casein kinase I isoform gamma-3;Casein kinase I isoform gamma-2;Casein kinase I isoform gamma-1	Csnk1g3;Csnk1g2;Csnk1g1	2.18656E-08	6.81128E-05
Cleavage stimulation factor subunit 2	Cstf2	1.08194E-08	0.000374016

Protein names	Gene names	Ratio (D2-T61I / D2-WT)	P-value
Cathepsin B;Cathepsin B light chain;Cathepsin B heavy chain	Ctsb	0.835886005	0.0402536
Cathepsin L1;Cathepsin L1 heavy chain;Cathepsin L1 light chain	Ctsl	0.74482533	0.044718684
Cullin-4B	Cul4b	0.81629355	0.017061135
UPF0606 protein KIAA1549	D630045J12Rik;Kiaa1549	2.02375E-08	3.40006E-05
Disheveled-associated activator of morphogenesis 2	Daam2	2.47001E-08	0.000303832
Disabled homolog 2-interacting protein	Dab2ip	4.482414366	0.018847481
Dystroglycan;Alpha-dystroglycan;Beta-dystroglycan	Dag1	0.231690318	0.037503257
Drebrin	Dbn1	1.445654287	0.033267036
Phospholipase DDHD2	Ddhd2	1.59686E-08	0.04576849
ATP-dependent RNA helicase DDX19B	Ddx19b	0.6567194	0.0249396
Protein DEK	Dek	1.389077517	0.029931598
DIS3-like exonuclease 2	Dis3l2	1.25372E-08	6.09723E-05
Dynamin-1	Dnm1	0.617365945	9.03917E-05
Double C2-like domain-containing protein alpha	Doc2a	59182333.33	2.3012E-06
Dual specificity protein phosphatase 3	Dusp3	0.714084972	0.03013467
Delta(3,5)-Delta(2,4)-dienoyl-CoA isomerase, mitochondrial	Ech1	3.37434576	0.011485798
Enoyl-CoA hydratase, mitochondrial	Echs1	1.261612339	0.044254915
Protein EFR3 homolog A	Efr3a	35048500	6.7633E-06
Eukaryotic translation initiation factor 2 subunit 2	Eif2s2	1.495063215	0.023721332
Eukaryotic translation initiation factor 3 subunit D	Eif3d	1.284993951	0.04281762
Eukaryotic initiation factor 4A-I	Eif4a1	1.164846775	0.025472135
Alpha-endosulfine	Ensa	1.403999482	0.011724283
Exocyst complex component 8	Exoc8	1.241630817	0.002703705
Constitutive coactivator of PPAR-gamma-like protein 1	FAM120A	0.881847346	0.041596207
F-box only protein 3	Fbxo3	3.92072E-09	0.025538761
Four and a half LIM domains protein 1	Fhl1	1.384693232	0.027867901
Acidic fibroblast growth factor intracellular-binding protein	Fibp	4.146766586	0.0321829
Peptidyl-prolyl cis-trans isomerase FKBP1B;Peptidyl-prolyl cis-trans isomerase	Fkbp1b	79684666.67	3.42215E-06
2-amino-3-ketobutyrate coenzyme A ligase, mitochondrial	Gcat	1.391947775	0.031796408
Glycerophosphodiester phosphodiesterase domain-containing protein 1	Gdpd1	1.396491373	0.044367887
GDP-mannose 4,6 dehydratase	Gmds	1.60366E-08	0.000534938
Guanine nucleotide-binding protein G(i) subunit alpha-1	Gnai1	0.822892028	0.024229203
Guanine nucleotide-binding protein G(olf) subunit alpha	Gnal	1.506177434	0.037991856

Protein names	Gene names	Ratio (D2-T61I / D2-WT)	P-value
Vesicle transport protein GOT1B	Golt1b	3.91988E-08	1.94939E-06
Prosaposin receptor GPR37	Gpr37	89704333.33	0.020982138
Metabotropic glutamate receptor 1	Grm1	2.6524E-08	0.000239252
Eukaryotic peptide chain release factor GTP-binding subunit ERF3A	Gspt1	0.826193699	0.033231404
Hydroxyacylglutathione hydrolase, mitochondrial	Hagh	0.795227019	0.038995822
Cytochrome c-type heme lyase	Hccs	48527000	0.01401886
HEAT repeat-containing protein 5B	Heatr5b	50201000	0.003361696
Heme-binding protein 1	Hebp1	1.251651642	0.026258693
Beta-hexosaminidase subunit alpha	Hexa	0.716324991	0.033102284
Huntingtin-interacting protein 1-related protein	Hip1r	1.13020744	0.002838472
Histone H1.1	Hist1h1a	1.423265534	0.032084905
Heterogeneous nuclear ribonucleoproteins A2/B1	Hnrnpa2b1	1.078561978	0.027593423
Heterogeneous nuclear ribonucleoproteins C1/C2	Hnrnpc	1.13278467	0.022810591
Heterogeneous nuclear ribonucleoprotein U-like protein 1	Hnrnpul1	1.560938603	0.016022168
Homer protein homolog 1	Homer1	0.768208177	0.049354281
Heterochromatin protein 1-binding protein 3	Hp1bp3	1.255704067	0.020091995
Hippocalcin-like protein 1	Hpcal1	1.514112451	0.027213094
Hydroxysteroid dehydrogenase-like protein 2	Hsdl2	1.511806954	0.014852436
Heat shock 70 kDa protein 12B	Hspa12b	2.65397E-09	1.0449E-08
10 kDa heat shock protein, mitochondrial	Hspe1;Cpn10-rs1	1.193736943	0.001614555
Isoamyl acetate-hydrolyzing esterase 1 homolog	Iah1	53740750	2.53466E-05
Insulin-degrading enzyme	Ide	0.656047727	0.010504302
Acetolactate synthase-like protein	Ilvbl	1.58095E-08	7.62394E-06
Protein IMPACT	Impact	1.287059685	0.023144563
Interactor protein for cytohesin exchange factors 1	Ipcef1	0.257877737	0.048417059
Iron-sulfur cluster assembly 1 homolog, mitochondrial	Isca1;AK157302	1.691351229	0.01279438
Isovaleryl-CoA dehydrogenase, mitochondrial	Ivd	1.355211071	0.013758176
Junctional adhesion molecule C	Jam3	1.272100488	0.02172714
Kelch repeat and BTB domain-containing protein 11	Kbtbd11	1.24116078	0.037366882
Potassium voltage-gated channel subfamily A member 4	Kcna4	1.9235E-08	0.000603575
Voltage-gated potassium channel subunit beta-2	Kcnab2	0.782341958	0.028828573
KH domain-containing, RNA-binding, signal transduction-associated protein 1	Khdrbs1	1.569447955	0.002872267
Far upstream element-binding protein 2	Khsrp	1.214253976	0.014147041

**Protein names**	**Gene names**	**Ratio (D2-T61I / D2-WT)**	**P-value**
GTPase KRas;GTPase KRas, N-terminally processed	Kras	0.70213803	0.020834939
Neural cell adhesion molecule L1	L1cam	1.188436951	0.036015977
Lysosome-associated membrane glycoprotein 2	Lamp2	0.201557695	0.024648138
Ragulator complex protein LAMTOR3	Lamtor3	5.04236E-09	8.18787E-06
La-related protein 1	Larp1	1.419552037	0.011265087
LIM and calponin homology domains-containing protein 1	Limch1	44007666.67	0.002472082
LMBR1 domain-containing protein 2	Lmbrd2	1.61785E-08	3.94792E-06
O-acetyl-ADP-ribose deacetylase MACROD1	Macrod1	1.561139595	0.039592928
Dual specificity mitogen-activated protein kinase kinase 1	Map2k1	1.264126655	0.039015649
Microtubule-associated protein 6	Map6	1.266831199	0.012662859
MAP7 domain-containing protein 2	Map7d2	1.460132281	0.041328783
Microtubule-associated protein RP/EB family member 3	Mapre3	1.220675523	0.002712413
Microtubule-associated protein tau;Microtubule-associated protein	Mapt	1.893844781	0.017946571
Myristoylated alanine-rich C-kinase substrate	Marcks	1.446976072	0.025861849
Microtubule-associated serine/threonine-protein kinase 1	Mast1	37354500	3.86434E-06
Methylcrotonoyl-CoA carboxylase subunit alpha, mitochondrial	Mccc1	1.643338438	0.004633743
Methylcrotonoyl-CoA carboxylase beta chain, mitochondrial	Mccc2	1.339384256	0.024846702
Malate dehydrogenase, cytoplasmic	Mdh1	1.247205727	0.007042561
NADP-dependent malic enzyme, mitochondrial	Me3	0.808355	0.03281876
Mitofusin-2	Mfn2	1.183187698	0.046895182
Macrophage migration inhibitory factor	Mif	1.496871115	0.025567264
MOB kinase activator 1A;MOB kinase activator 1B	Mob1b;Mob1a	47996000	0.0009926
Adenylyltransferase and sulfurtransferase MOCS3;Molybdopterin-synthase adenylyltransferase;Molybdopterin-synthase sulfurtransferase	Mocs3	50124000	0.000146671
Mitochondrial pyruvate carrier 1	Mpc1	0.748152812	0.026771102
39S ribosomal protein L2, mitochondrial	Mrpl2	0.149512138	0.025838837
39S ribosomal protein L9, mitochondrial	Mrpl9	3.63897E-08	7.27632E-05
28S ribosomal protein S5, mitochondrial	Mrps5	2.15123E-08	0.000116571
C-1-tetrahydrofolate synthase, cytoplasmic;Methylenetetrahydrofolate dehydrogenase;Methenyltetrahydrofolate cyclohydrolase;Formyltetrahydrofolate synthetase;C-1-tetrahydrofolate synthase, cytoplasmic, N-terminally processed	Mthfd1	1.237242693	0.014506508

Protein names	Gene names	Ratio (D2-T61I / D2-WT)	P-value
Metaxin-3	Mtx3	0.240321478	0.039150583
N-alpha-acetyltransferase 15, NatA auxiliary subunit	Naa15	1.275013316	0.013845446
Nucleosome assembly protein 1-like 5	Nap1l5	1.70722E-08	0.006134588
Neural cell adhesion molecule 1	Ncam1	1.768834203	0.041778615
Cytoplasmic protein NCK2	Nck2	0.874650187	0.026103855
Nicastrin	Ncstn	1.32764E-08	0.000394093
Adaptin ear-binding coat-associated protein 1	Necap1	1.261497515	0.040318995
Nischarin	Nisch	1.155319238	0.023244812
Nucleoside diphosphate kinase A;Nucleoside diphosphate kinase	Nme1	0.487075764	0.017862547
RNA-binding protein Nova-1	Nova1	64517250	0.010547938
5(3)-deoxyribonucleotidase, mitochondrial	Nt5m	4.07567703	0.025569852
Diphosphoinositol polyphosphate phosphohydrolase 3-beta;Diphosphoinositol polyphosphate phosphohydrolase 3-alpha	Nudt11;Nudt10	1.554335667	0.005939545
Cleavage and polyadenylation specificity factor subunit 5	Nudt21	0.798498008	0.045718908
Nuclear transport factor 2	Nutf2	1.108721332	0.032827006
3-oxoacyl-[acyl-carrier-protein] synthase, mitochondrial	Oxsm	64030000	0.00081922
Polyadenylate-binding protein	Pabpc4	0.380527813	0.018539344
Pantothenate kinase 4	Pank4	1.383735935	0.038426543
Protocadherin-9	Pcdh9	2.21517E-08	0.02365912
ProSAAS;KEP;Big SAAS;Little SAAS;Big PEN-LEN;PEN;PEN-20;PEN-19;Little LEN;Big LEN	Pcsk1n	105825000	0.000969669
cAMP and cAMP-inhibited cGMP 3,5-cyclic phosphodiesterase 10A	Pde10a	1.209593973	0.04192034
Peflin	Pef1	1.410910776	0.011093059
Prefoldin subunit 2	Pfdn2	1.46099845	0.030591532
Phosphoglucomutase-1	Pgm1;Pgm2	1.234921401	0.021017152
14 kDa phosphohistidine phosphatase	Phpt1	1.291099656	0.008643456
Membrane-associated phosphatidylinositol transfer protein 3	Pitpnm3	1.58664E-08	0.002233616
Plasmolipin	Pllp	0.490855262	0.0164545
Plexin-B2	Plxnb2	46717000	8.53528E-05
Phosphomannomutase 2	Pmm2	0.188611592	0.023846324
Calcium/calmodulin-dependent protein kinase type 1B	Pnck	48538333.33	0.004574932
Peptidyl-prolyl cis-trans isomerase F, mitochondrial	Ppif	8.26221E-09	0.001178387
Protein phosphatase 1F	Ppm1f	1.19175024	0.049402724
Protein phosphatase 1 regulatory subunit 12A	Ppp1r12a	1.20289632	0.032126972

Protein names	Gene names	Ratio (D2-T61I / D2-WT)	P-value
Neurabin-2	Ppp1r9b	1.723508716	0.027791606
Serine/threonine-protein phosphatase 2A 56kDa regulatory subunit be	Ppp2r5b	80689500	0.002986298
Serine/threonine-protein phosphatase 2B catalytic subunit alpha isofor	Ppp3ca	0.830476764	0.016317885
Peroxiredoxin-1	Prdx1	1.261506216	0.009392857
Peroxiredoxin-2	Prdx2	1.266832809	0.016779034
Protein kinase C beta type	Prkcb	0.88384926	0.026142273
Interferon-inducible double-stranded RNA-dependent protein kinase a	Prkra	1.759322751	0.013049816
Ribose-phosphate pyrophosphokinase 1	Prps1l3;Prps1	1.237678729	0.012075501
26S protease regulatory subunit 8	Psmc5	1.118819808	0.033446225
26S proteasome non-ATPase regulatory subunit 7	Psmd7	1.308399546	0.04473828
Prostaglandin-H2 D-isomerase	Ptgds	0.628439137	0.028650368
Focal adhesion kinase 1	Ptk2	0.803158567	0.039365623
Protein tyrosine phosphatase type IVA 1	Ptp4a1	40426666.67	0.002884972
Phosphatidylglycerophosphatase and protein-tyrosine phosphatase 1	Ptpmt1	0.836711256	0.047753454
Transcriptional activator protein Pur-beta	Purb	0.720829284	0.023107956
Ras-related protein Rab-39A	Rab39a	6.77767E-09	0.000486199
Ras-related protein Rab-39B	Rab39b	1.533976025	0.034872026
Ras-related C3 botulinum toxin substrate 3	Rac3	1.26759E-08	2.88578E-06
Rap1 GTPase-activating protein 2	Rap1gap2	0.867049827	0.001861638
Rap guanine nucleotide exchange factor 4	Rapgef4	0.647459861	0.019917569
RNA binding protein fox-1 homolog 1;RNA binding protein fox-1 homolog 2	Rbfox1;Rbfox2	0.739062516	0.037543548
RNA-binding protein 8A	Rbm8a	0.737956966	0.027319025
Calcipressin-1	Rcan1	57219666.67	0.000392179
Riboflavin kinase	Rfk	42127000	0.003215651
Regulating synaptic membrane exocytosis protein 3	Rims3	1.32696E-08	0.000566736
Ras and Rab interactor 1	Rin1	0.685209169	0.029356294
Ribonuclease inhibitor	Rnh1	1.173316983	0.017570646
Rabphilin-3A	Rph3a	0.847317005	0.023552453
60S ribosomal protein L13a	Rpl13a	1.42155436	0.023957632
60S ribosomal protein L14	Rpl14	1.415507512	0.021069253
60S ribosomal protein L17	Rpl17	1.547967654	0.012173427
60S ribosomal protein L3	Rpl3	1.354521328	0.041722413
60S ribosomal protein L5	Rpl5	1.352177467	0.033860159

Protein names	Gene names	Ratio (D2-T61I / D2-WT)	P-value
40S ribosomal protein S17	Rps17	1.271583466	0.049981649
40S ribosomal protein S2	Rps2	1.37051848	0.004353685
40S ribosomal protein S21	Rps21	1.369095297	0.033305643
40S ribosomal protein S7	Rps7	1.166058149	0.039834124
Regulatory-associated protein of mTOR	Rptor	1.370466734	0.046868564
Ras-related GTP-binding protein A	Rraga	1.229160024	0.036999886
Reticulon-3	Rtn3	4.874971599	0.015211689
RuvB-like 2	Ruvbl2	1.265653088	0.047492016
Protein S100;Protein S100-A1	S100a1	1.201227153	0.037392185
Phosphatidylinositide phosphatase SAC1	Sacm1l	0.926069632	0.030630733
Secretory carrier-associated membrane protein 3	Scamp3	1.820147441	0.001442926
Secretogranin-2;Secretoneurin;Manserin	Scg2	1.897419799	0.030689988
Sodium channel subunit beta-2	Scn2b	0.712821242	0.034572063
Non-specific lipid-transfer protein	Scp2	1.282842694	0.021271025
Scrapie-responsive protein 1	Scrg1	40212333.33	4.67363E-06
Protein transport protein Sec61 subunit alpha isoform 1	Sec61a1	69226000	0.000464948
Neuroserpin	Serpini1	43177000	0.001084022
Histone-lysine N-methyltransferase setd3	Setd3	1.75269E-08	2.00618E-05
Splicing factor 3A subunit 1	Sf3a1	1.307723718	0.045793307
SH3-containing GRB2-like protein 3-interacting protein 1	Sgip1	1.201358962	0.036222256
Small glutamine-rich tetratricopeptide repeat-containing protein alpha	Sgta	1.253404044	0.041136847
Small glutamine-rich tetratricopeptide repeat-containing protein beta	Sgtb	0.756234637	0.038954549
Excitatory amino acid transporter 1	Slc1a3	1.521375445	0.023367269
sodium potassium calcium exchanger 2	Slc24a2	0.665714338	0.012312174
Sodium- and chloride-dependent GABA transporter 3	Slc6a11	1.785147207	0.018825345
Cystine/glutamate transporter	Slc7a11	7.890692806	0.004652403
Staphylococcal nuclease domain-containing protein 1	Snd1	1.229149937	0.006921953
U1 small nuclear ribonucleoprotein 70 kDa	Snrnp70	1.284852278	0.001951492
U1 small nuclear ribonucleoprotein C	Snrpc	4.517077383	0.013907421
Small nuclear ribonucleoprotein Sm D1	Snrpd1	0.672112249	0.031600463
Small nuclear ribonucleoprotein E	Snrpe	0.72135265	0.037365646
SNW domain-containing protein 1	Snw1	49978000	0.000941182
Sorting nexin-27	Snx27	1.304499339	0.03509189
Sorting nexin-30	Snx30	1.546646131	0.008266792

Protein names	Gene names	Ratio (D2-T61I / D2-WT)	P-value
Superoxide dismutase [Cu-Zn]	Sod1	1.380970542	0.028463429
Sorbin and SH3 domain-containing protein 1	Sorbs1	2.19956E-08	0.001556259
C-Jun-amino-terminal kinase-interacting protein 4	Spag9	1.346418372	0.024857555
Spermatogenesis-associated protein 2-like protein	Spata2l	2.49881E-08	0.004093083
Spondin-1	Spon1	3.43619E-08	0.000245429
Sepiapterin reductase	Spr	1.219855337	0.017731472
Serine/arginine-rich splicing factor 3	Srsf3	0.54199142	0.016948012
Synergin gamma	Synrg	1.385422337	0.02503123
Transaldolase	Taldo1	1.250506262	0.038419175
Tubulin-folding cofactor B	Tbcb	1.19384727	0.038767058
Tubulin-specific chaperone C	Tbcc	1.02941E-08	0.027526412
Tubulin-specific chaperone E	Tbce	1.57637E-08	0.000338055
Transcription elongation factor B polypeptide 1	Tceb1	1.508270657	0.00408039
Teneurin-4	Tenm4	96063000	0.00013302
Threonine synthase-like 1	Thnsl1	1.489419241	0.000226961
THUMP domain-containing protein 1	Thumpd1	3.797462427	0.048982949
Transmembrane protein 132B	Tmem132b	0.732987257	0.044333856
Mitochondrial import receptor subunit TOM34	Tomm34	1.312779379	0.015478412
Tropomyosin 1	Tpm1	1.752442127	0.018595274
Tubulin polymerization-promoting protein	Tppp	1.206934197	0.006705494
E3 ubiquitin-protein ligase TRIM9	Trim9	0.782333832	0.025285841
Tetraspanin-7;Tetraspanin	Tspan7	0.468638016	0.013668653
Pre-rRNA-processing protein TSR2 homolog	Tsr2	1.382226505	0.045635546
Tubulin alpha-1A chain;Tubulin alpha-3 chain	Tuba1a;Tuba3a	0.594047977	0.02597128
Tumor suppressor candidate 2	Tusc2	4.929051941	0.011052226
Twinfilin-1	Twf1	1.379061825	0.028863211
Thioredoxin domain-containing protein 9	Txndc9	4.125306679	0.040230403
Ubiquitin-conjugating enzyme E2 K	Ube2k	1.259119323	0.005709709
Ubiquitin-conjugating enzyme E2 variant 1	Ube2v1;Gm20431	0.227756394	0.028022069
Ubiquitin-protein ligase E3B	Ube3b	38569666.67	4.28226E-05
Up-regulated during skeletal muscle growth protein 5	Usmg5	0.671732935	0.04005365
Ubiquitin carboxyl-terminal hydrolase 11	Usp11	42494333.33	0.027260982
Ubiquitin carboxyl-terminal hydrolase;Ubiquitin carboxyl-terminal hydrolase 14	Usp14	1.195353258	0.035294731

**Protein names**	**Gene names**	**Ratio (D2-T61I / D2-WT)**	**P-value**
Synaptic vesicle membrane protein VAT-1 homolog-like	Vat1l	1.742070463	0.048910806
Nuclease-sensitive element-binding protein 1	Ybx1	2.46251E-08	0.019017046
14-3-3 protein beta/alpha;14-3-3 protein beta/alpha, N-terminally processed	Ywhab	1.398202716	0.018158031
14-3-3 protein epsilon	Ywhae	1.272940543	0.005393949
14-3-3 protein theta	Ywhaq	1.681652756	0.003172961
14-3-3 protein zeta/delta	Ywhaz	1.319284226	0.032417904
Zyxin	Zyx	3.078801065	0.021785053
Uncharacterized protein C2orf47 homolog, mitochondrial		171823333.3	0.01586514
Uncharacterized protein C1orf198 homolog		3.228485445	0.02111194

Table S2: Top 30 canonical pathways significantly altered in the cortex of D2-T61I vs D2-WT mice

Ingenuity Canonical Pathways	-Log(p-value)	Z-score	Ratio	Molecules
Fatty Acid β-oxidation I	5.15	2.449489743	0.182	ACSBG1,ACSL1,ACSL3,ECHS1,IVD,SCP2
EIF2 Signaling	4.85	1.889822365	0.0613	EIF2S2,EIF3D,EIF4A1,KRAS,MAP2K1,RPL13A,RPL14,RPL17,RPL3,RPL5,RPS2,RPS21,RPS7
p70S6K Signaling	4.33	-0.816496581	0.0699	GNAI1,KRAS,MAP2K1,MAPT,PPP2R5B,PRKCB,YWHAB,YWHAE,YWHAQ,YWHAZ
Virus Entry via Endocytic Pathways	4.25		0.0763	ACTG1,AP3B2,AP3M1,AP3S1,AP3S2,DNM1,KRAS,PRKCB,RAC3
VEGF Signaling	4.01	0	0.0808	ACTG1,ACTN4,EIF2S2,KRAS,MAP2K1,PRKCB,PTK2,YWHAE
CLEAR Signaling Pathway	3.66	-1.941450687	0.0469	CTSB,HEXA,KRAS,LAMTOR3,PPP2R5B,PPP3CA,PRKCB,RPTOR,RRAGA,YWHAB,YWHAE,YWHAQ,YWHAZ
ERK/MAPK Signaling	3.62	-1.889822365	0.0529	KRAS,LAMTOR3,MAP2K1,PPP2R5B,PRKCB,PTK2,RAC3,RAPGEF4,YWHAB,YWHAQ,YWHAZ
Leucine Degradation I	3.62		0.333	IVD,MCCC1,MCCC2
14-3-3-mediated Signaling	3.34	0.707106781	0.0645	KRAS,MAP2K1,MAPT,PRKCB,YWHAB,YWHAE,YWHAQ,YWHAZ
mTOR Signaling	3.12	-1	0.0495	EIF3D,EIF4A1,KRAS,PPP2R5B,PRKCB,RAC3,RPS2,RPS21,RPS7,RPTOR
IGF-1 Signaling	3.09		0.0673	KRAS,MAP2K1,PTK2,YWHAB,YWHAE,YWHAQ,YWHAZ
Regulation of eIF4 and p70S6K Signaling	3.01		0.052	EIF2S2,EIF3D,EIF4A1,KRAS,MAP2K1,PPP2R5B,RPS2,RPS21,RPS7
Fatty Acid Activation	2.92		0.2	ACSBG1,ACSL1,ACSL3
Isoleucine Degradation I	2.75		0.176	ACAD8,ACADSB,ECHS1
Mitochondrial L-carnitine Shuttle Pathway	2.61		0.158	ACSBG1,ACSL1,ACSL3
γ-linolenate Biosynthesis II (Animals)	2.61		0.158	ACSBG1,ACSL1,ACSL3
Clathrin-mediated Endocytosis Signaling	2.59		0.0452	ACTG1,AP3B2,AP3M1,AP3S1,AP3S2,APOE,DNM1,HIP1R,PPP3CA
fMLP Signaling in Neutrophils	2.55	-1.341640786	0.0543	GNAI1,GNAL,KRAS,MAP2K1,PPP3CA,PRKCB,RAC3
Valine Degradation I	2.54		0.15	ACAD8,ACADSB,ECHS1
Netrin Signaling	2.46	-1.341640786	0.0714	ABLIM1,CACNB3,NCK2,PPP3CA,RAC3
Integrin Signaling	2.46	-1.133893419	0.0433	ACTG1,ACTN4,KRAS,MAP2K1,NCK2,PTK2,RAC3,TSPAN7,ZYX
Ephrin B Signaling	2.4	-1.341640786	0.0694	GNAI1,GNAL,NCK2,PTK2,RAC3
ERK5 Signaling	2.35	1.341640786	0.0676	KRAS,YWHAB,YWHAE,YWHAQ,YWHAZ

Ingenuity Canonical Pathways	-Log(p-value)	Z-score	Ratio	Molecules
Paxillin Signaling	2.33	-1.632993162	0.0561	ACTG1,ACTN4,ARFIP2,KRAS,NCK2,PTK2
NRF2-mediated Oxidative Stress Response	2.33	1.133893419	0.0413	ACTG1,AKR1A1,KRAS,MAP2K1,PRDX1,PRKCB,SOD1,UBE2K,USP14
Protein Kinase A Signaling	2.31	1.386750491	0.0332	DUSP3,GDPD1,GNAI1,MAP2K1,PDE10A,PPP3CA,PRKCB,PTK2,PTP4A1,YWHAB,YWHAE,YWHAQ,YWHAZ
Chemokine Signaling	2.28	-1.341640786	0.0649	GNAI1,KRAS,MAP2K1,PRKCB,PTK2
Synaptic Long Term Depression	2.16	-1.414213562	0.0419	CACNB3,GNAI1,GNAL,GRM1,KRAS,MAP2K1,PPP2R5B,PRKCB
Glutaminergic Receptor Signaling Pathway (Enhanced)	2.16	-0.904534034	0.0347	CACNB3,GNAI1,GNAL,GRM1,HOMER1,PPP3CA,PRKCB,RPTOR,SCN2B,SLC1A3,SLC7A11
HIPPO signaling	2.1	-2.236067977	0.0588	PPP2R5B,YWHAB,YWHAE,YWHAQ,YWHAZ
